# Efficacy and safety of botulinum toxin type a for upper-limb essential tremor: a systematic review and meta-analysis of randomized placebo-controlled trials

**DOI:** 10.3389/fneur.2026.1886329

**Published:** 2026-08-28

**Authors:** Guorui Wang, Liping Cheng, Ziwei Liao

**Affiliations:** 1 Department of Clinical Test Labs, Handan First Hospital, Handan, Hebei, China; 2 Department of Second Neurology, Handan First Hospital, Handan, Hebei, China

**Keywords:** botulinum toxin type A, essential tremor, GRADE, meta-analysis, muscular weakness, systematic review, upper-limb tremor

## Abstract

**Background:**

Botulinum toxin type A (BoNT-A) is used as a targeted treatment for upper-limb essential tremor, but uncertainty remains regarding the magnitude and certainty of its benefits and harms. We evaluated the efficacy and safety of BoNT-A compared with placebo in adults with upper-limb essential tremor.

**Methods:**

We systematically searched bibliographic databases, trial registries, and eligible grey-literature sources from inception to July 20, 2026. We included randomized placebo-controlled trials of BoNT-A in adults with upper-limb essential tremor. Primary outcomes were short-term clinician-rated upper-limb tremor severity and muscular weakness through Week 24. Continuous outcomes were summarized using mean differences or Hedges’ g, and binary safety outcomes using risk differences. Random-effects meta-analyses were performed. Risk of bias was assessed at the result level using the Cochrane Risk of Bias 2 tool, and certainty of evidence was evaluated using GRADE.

**Results:**

Three trials with outcome data from 231 participants contributed to the primary efficacy analysis. BoNT-A reduced short-term clinician-rated upper-limb tremor severity compared with placebo (Hedges’ g, −0.66; 95% CI, −0.94 to −0.38; I^2^ = 0%; moderate-certainty evidence). Muscular weakness was more frequent with BoNT-A (2 trials; 107 participants; risk difference, 0.19; 95% CI, 0.06 to 0.32; I^2^ = 29%; low-certainty evidence), approximately 190 more participants per 1,000 treated. BoNT-A may improve investigator-rated global treatment response (Hedges’ g, 0.70; 95% CI, 0.06 to 1.34) and patient-rated global treatment response (Hedges’ g, 0.40; 95% CI, 0.14 to 0.67), although certainty was low. Maximum grip strength was reduced (mean difference, −7.13 kg; 95% CI, −11.42 to −2.85; low-certainty evidence). Evidence regarding treatment-related adverse events was very uncertain, whereas estimates for serious adverse events and discontinuation due to adverse events were too imprecise to exclude clinically important benefit or harm.

**Conclusion:**

BoNT-A probably reduces short-term clinician-rated upper-limb tremor severity but may increase muscular weakness and reduce grip strength. Evidence for global treatment response and less frequent adverse outcomes remains limited or very uncertain. Treatment decisions should balance the expected reduction in tremor against the individualized risk of weakness and functional impairment.

## 1 Introduction

Essential tremor (ET) is currently classified as an isolated tremor syndrome characterized principally by bilateral upper-limb action tremor of at least 3 years’ duration, with or without tremor in other anatomical locations, and without other neurological signs such as dystonia, ataxia, or parkinsonism (1). This definition distinguishes ET from other tremor syndromes, including dystonic tremor, functional tremor, orthostatic tremor, parkinsonian rest tremor, and isolated focal tremors (1). The distinction is clinically important because these syndromes differ in pathophysiology, treatment response, and prognosis (2, 3). ET can progressively interfere with writing, drinking, eating, dressing, and other activities requiring fine motor control (2, 4).

Propranolol and primidone are commonly used oral treatments for ET, but their effectiveness and tolerability vary between patients (5, 6). Other pharmacological and surgical options may be considered according to tremor severity, anatomical distribution, comorbidity, and patient preference (3, 7, 8). BoNT-A provides a targeted peripheral treatment by weakening selected muscles involved in tremor generation; however, the same mechanism may cause focal weakness and functional impairment (9, 10). Existing evidence reviews have therefore generally regarded BoNT-A as a possible treatment option in selected patients rather than as an established first-line therapy (7, 8, 11).

Previous systematic reviews have reached differing conclusions regarding the benefits and harms of BoNT-A for ET (11–13). Differences between reviews may reflect variation in diagnostic populations, anatomical tremor distribution, injection strategies, outcome selection, follow-up time points, and the handling of treatment-related weakness (9, 11, 13). More recent randomized evidence has also included populations with head tremor and emerging upper-limb trial data, requiring renewed assessment of study eligibility and clinical comparability (14–20).

The objective of this systematic review and meta-analysis was to evaluate the efficacy and safety of BoNT-A compared with placebo in adults with upper-limb essential tremor.

## 2 Methods

### 2.1 Protocol and reporting

This systematic review and meta-analysis was reported in accordance with the Preferred Reporting Items for Systematic Reviews and Meta-Analyses (PRISMA) 2020 statement (21). The review was not prospectively registered, and no prospectively published protocol was available. In response to peer review, we revised the eligibility criteria, outcome hierarchy, analytical methods, sensitivity analyses, RoB 2 procedures, and GRADE thresholds. Analyses introduced during revision are identified as amended or exploratory. A completed PRISMA 2020 checklist is provided as Supplementary material.

### 2.2 Eligibility criteria

Eligibility criteria were structured according to the Participants, Intervention, Comparator, Outcomes, and Study design (PICOS) framework:

(1) Participants (P): We included adults aged 18 years or older diagnosed with essential tremor by the original trial investigators, provided that upper-limb action tremor was the anatomical target of treatment. Trials targeting isolated head tremor, or mixed isolated and essential head tremor without separately extractable upper-limb essential-tremor data, were excluded. Sex and ethnicity were not used as eligibility restrictions but were extracted when reported.
(2) Intervention (I): Eligible interventions consisted of intramuscular injection of any formulation of botulinum toxin type A (BoNT-A), irrespective of total dose, muscle-selection strategy, injection-guidance technique, number of injected muscles, or number of treatment cycles.
(3) Comparator (C): The eligible comparator was placebo or saline injection administered under otherwise comparable trial conditions. Trials without a placebo or saline comparator, including active-comparator trials and comparisons of alternative BoNT-A injection strategies, were excluded.
(4) Outcomes (O): Trials were required to report at least one eligible efficacy, functional, or safety outcome. The primary efficacy outcome was short-term clinician-rated upper-limb tremor severity. The primary safety outcome was muscular weakness during randomized follow-up through Week 24. Secondary outcomes were investigator-rated global treatment response, patient-rated global treatment response, maximum grip strength, functional or activities-of-daily-living outcomes, treatment-related treatment-emergent adverse events, serious adverse events, and discontinuation due to adverse events.
(5) Study design (S): We included randomized placebo-controlled trials with parallel-group or crossover designs. Crossover trials were eligible for quantitative synthesis only when a paired treatment effect with adequate variance information, sufficient within-participant data, or separable first-period data was available. Treatment-period observations were not treated as independent parallel-group observations.

We excluded nonrandomized studies, uncontrolled studies, observational studies, case reports, reviews, editorials, animal studies, and duplicate reports. Lack of open access or insufficient data for meta-analysis was not itself an exclusion criterion. When multiple reports described the same trial, they were linked and treated as a single study. Eligible studies with insufficient data for appropriate quantitative synthesis were retained for narrative synthesis.

### 2.3 Search methods

We searched MEDLINE via PubMed and Ovid, Embase, the Cochrane Central Register of Controlled Trials via the Cochrane Library, Web of Science Core Collection, ProQuest Dissertations & Theses Global, CNKI, WanFang Data, and SinoMed from inception to July 20, 2026. ClinicalTrials.gov and the WHO International Clinical Trials Registry Platform were searched for completed, ongoing, and unpublished trials. No language restrictions were applied.

Backward reference checking was conducted for included studies and relevant reviews. Forward citation searching was restricted to eligible or included upper-limb placebo-controlled trial reports. Conference abstracts, dissertations, trial-registry records, and publicly available sponsor sources were also considered. The search combined controlled vocabulary and free-text terms relating to essential tremor, upper-limb or hand tremor, botulinum toxin type A, randomization, and placebo. Database platforms, database-specific search dates, complete search strategies, and numbers of records retrieved are provided in Supplementary Table S1.

### 2.4 Study selection

Two reviewers independently screened titles and abstracts and subsequently assessed potentially eligible full-text reports. Each record was classified as included, excluded, awaiting clarification, or linked to another report of the same study. Disagreements were resolved through discussion and, when necessary, consultation with a third reviewer.

**Figure 1:**
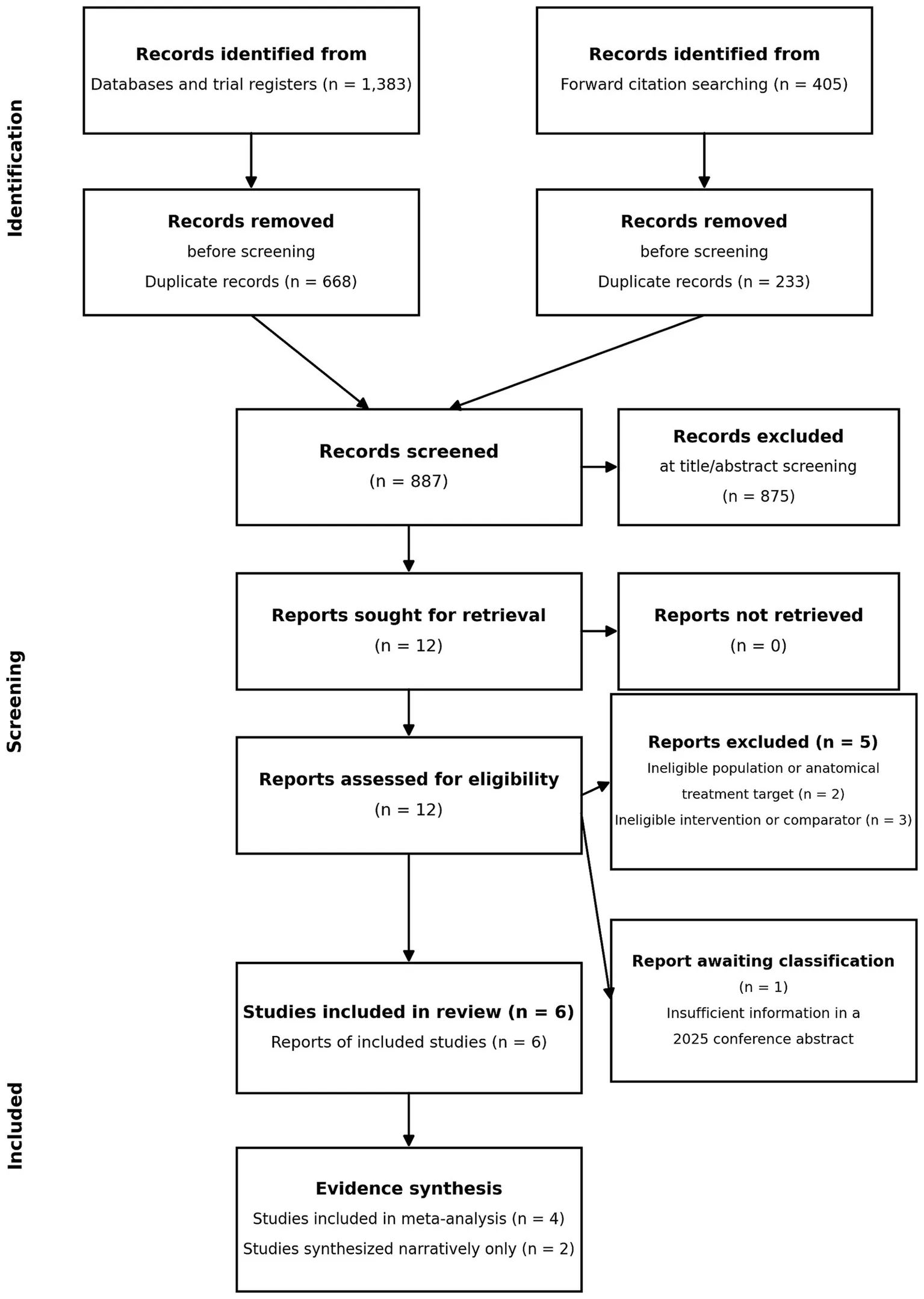
PRISMA 2020 flow diagram. The diagram shows records identified, duplicate records removed, records screened, reports sought and assessed for eligibility, exclusions with reasons, and studies included in the review and quantitative synthesis.

Reports excluded after detailed eligibility assessment, together with the report awaiting classification, are listed in Supplementary Table S2. For the PRISMA flow diagram, one principal eligibility report was counted for each included study; additional linked registry, conference, and sponsor sources used for design or result verification are listed in Supplementary Table S3 and were not counted as separate eligibility reports. Records, reports, and studies were distinguished in accordance with PRISMA 2020 terminology. The study-selection process is shown in Figure 1.

### 2.5 Data extraction and verification

Two reviewers independently extracted and verified study-level and result-level data using a standardized form. Study characteristics included publication year, country, diagnostic criteria, study design, participant age, sex, ethnicity when reported, tremor distribution, disease duration, toxin formulation, total dose, injected muscles, muscle-selection strategy, injection guidance, treatment schedule, comparator, outcome instruments, assessment times, and duration of randomized follow-up.

For each quantitative result, we extracted the outcome definition, rater, anatomical site, assessment time, analysis population, intervention and control group sizes, means, standard deviations, endpoint or change-score status, event counts, effect estimates, confidence intervals, and exact source location.

Data were checked against journal articles, trial-registry records, protocols, statistical analysis plans, supplementary materials, conference reports, and sponsor-reported results when available. Discrepancies were resolved by returning to the primary source rather than relying on values reported in previous reviews. Study-to-report linkage and result-level source data, including denominators, numerical values, derivations, and source locations, are provided in Supplementary Table S3. Additional linked registry, conference, and sponsor sources used for verification are distinguished from the six principal included reports counted in Figure 1.

### 2.6 Outcome definitions and data-selection hierarchy

The primary efficacy outcome was clinician-rated tremor severity of the treated upper limb at the eligible assessment closest to 4–6 weeks after the first randomized injection. Eligible measures included validated clinician-rated scales, such as the Fahn–Tolosa–Marín Tremor Rating Scale and TETRAS, and clearly defined trial-specific clinician ratings of postural or kinetic tremor. Scale directions were harmonized so that negative standardized effects indicated benefit from BoNT-A.

Investigator-rated and patient-rated global treatment responses were analyzed separately because they represented different raters and potentially different constructs. They were not combined into a common overall estimate, and no subgroup-difference test assuming independence between the two rater groups was performed.

Brin (2001) assessed global response relative to the preceding visit, whereas the other studies assessed response relative to baseline or overall treatment status. Its measures were judged to represent the same broad construct of short-term global treatment response and were retained in the primary analyses. Sensitivity analyses excluding Brin (2001) were conducted to assess the robustness of the pooled investigator- and patient-rated global-response estimates to its different reference frame.

Maximum grip strength was analyzed in kilograms, with lower values indicating greater loss of strength. Binary safety outcomes were defined as the number of participants experiencing at least one event during the eligible randomized follow-up.

When multiple eligible measures were available for the same outcome, selection followed the revised outcome hierarchy described above rather than statistical significance. The trial-designated primary or most directly relevant measure was preferred, followed by the assessment closest to the eligible time window. Alternative eligible measurements were examined in sensitivity analyses. Study-specific responder outcomes were not pooled unless their definitions, thresholds, raters, time points, and statistical structures were sufficiently comparable.

### 2.7 Handling of multiple reports, multiple treatment arms, and crossover trials

Multiple reports from the same underlying trial were linked to prevent double-counting of participants.

When a parallel-group trial included more than one eligible BoNT-A group sharing a common placebo group, the active-treatment groups were combined into one comparison. Means were combined using sample-size weighting, and standard deviations were combined using the standard formula incorporating within-group variance and differences between group means, following current Cochrane guidance (22).

Crossover trials were not analyzed as if treatment-period observations were independent. Paired estimates or methods accounting for within-participant correlation were used when available. When paired data were unavailable, separable first-period data were sought. Eligible crossover trials without adequate paired or first-period information were summarized narratively. Period effects, carryover effects, treatment-sequence balance, and adequacy of washout were considered in the crossover-specific risk-of-bias assessment.

### 2.8 Risk-of-bias assessment

Two reviewers independently assessed risk of bias for each eligible result using the Cochrane Risk of Bias 2 tool (23). Parallel-group results were assessed for bias arising from the randomization process, deviations from intended interventions, missing outcome data, outcome measurement, and selection of the reported result.

Crossover-trial results were assessed using the RoB 2 crossover variant, including the additional domain concerning period and carryover effects. Overall judgments were categorized as low risk of bias, some concerns, or high risk of bias.

Particular attention was given to potential functional unblinding caused by recognizable treatment-related weakness. For trials without accessible protocols or statistical analysis plans, the availability of multiple eligible measures, time points, or analyses was considered when assessing selective reporting. Disagreements were resolved by consensus.

### 2.9 Data synthesis and statistical analysis

Continuous outcomes reported on the same scale were summarized using mean differences. When studies assessed the same clinical construct using different instruments, effects were expressed as standardized mean differences. Hedges’ g was used because several trials had small sample sizes and the correction reduces the small-sample upward bias of Cohen’s d. Binary safety outcomes were summarized using risk differences to facilitate interpretation of absolute effects. All estimates are reported with 95% confidence intervals.

Endpoint and change scores were combined only in standardized-effect analyses when they represented the same clinical construct and had compatible scale direction. Endpoint and change scores were not mixed in mean-difference analyses.

Random-effects models were used because clinical diversity was expected. For continuous outcomes, between-study variance was estimated using restricted maximum likelihood when at least three studies were available. When at least three studies were included and the estimated between-study variance was greater than zero, 95% confidence intervals were calculated using the modified Hartung–Knapp method (34); otherwise, Wald-type confidence intervals were used. Sparse binary outcomes were pooled as Mantel–Haenszel random-effects risk differences.

Statistical heterogeneity was described using τ^2^, Cochran’s Q, and I^2^. Because each analysis contained few studies, heterogeneity was interpreted using the magnitude and direction of study estimates, overlap of confidence intervals, clinical characteristics, and sensitivity analyses rather than fixed I^2^ thresholds alone.

Review Manager 5.3 was used for data management and eligible two-study or zero-heterogeneity analyses. Stata version 15.1 was used for restricted-maximum-likelihood and modified Hartung–Knapp analyses.

Formal funnel-plot asymmetry and regression tests were not performed because no synthesis contained enough studies to provide reliable results. Potential publication bias was considered qualitatively by comparing published reports with trial registrations, protocols, statistical analysis plans, and available unpublished, sponsor-, or conference-reported evidence.

### 2.10 Sensitivity analyses

For clinician-rated upper-limb tremor severity, sensitivity analyses substituted the alternative Brin kinetic-tremor measure, excluded Brin (2001), and added the converted Jankovic responder result. The responder conversion was considered exploratory because a binary response outcome was transformed into a standardized continuous effect. Investigator- and patient-rated global-response analyses were repeated after excluding Brin (2001). For maximum grip strength, the alternative eligible Brin dynamometer measurement was substituted.

Sensitivity analyses were interpreted according to changes in effect direction, magnitude, confidence-interval width, and heterogeneity rather than statistical significance alone. For the exploratory analysis, the odds ratio derived from the Jankovic dichotomous responder data was converted to an approximate standardized mean difference using SMD = ln(OR) × √3/*π*, with the sign oriented so that negative values favored BoNT-A (24). The complete sensitivity analyses and outcomes not included in pooled estimates are summarized in Supplementary Table S4.

### 2.11 Certainty of evidence

Two reviewers independently assessed certainty of evidence for each principal outcome using GRADE (25–27). Randomized evidence started at high certainty and was downgraded for risk of bias, inconsistency, indirectness, imprecision, or publication bias.

Imprecision was evaluated using review-specific decision thresholds, confidence-interval width, sample size, and event numbers. The thresholds were 0.20 standardized-deviation units for standardized outcomes, 5 kg for grip strength, 5 percentage points for muscular weakness, 10 percentage points for treatment-related treatment-emergent adverse events, 2 percentage points for serious adverse events, and 5 percentage points for discontinuation due to adverse events. These were review-specific decision thresholds rather than validated essential-tremor-specific minimal important differences.

A complete GRADE Evidence Profile is provided in Supplementary Table S5. A Summary of Findings table was prepared for the seven outcomes judged most important for clinical decision-making. Absolute binary effects were expressed per 1,000 participants using the aggregate placebo risk and pooled risk difference.

## 3 Results

### 3.1 Study selection

The searches identified 1,788 records. After removal of 901 duplicates, 887 unique records were screened, and 875 were excluded at title and abstract screening. Twelve principal reports were sought for retrieval; all were obtained and assessed for eligibility. Five reports were excluded after detailed assessment (ineligible population or anatomical treatment target, *n* = 2; ineligible intervention or comparator, *n* = 3), and one 2025 conference abstract remained awaiting classification because the available information was insufficient to determine eligibility. Six principal reports, each corresponding to one of six randomized placebo-controlled studies, were included in the review; four studies contributed to at least one quantitative synthesis and two were summarized narratively (Figure 1).

### 3.2 Study characteristics

The six studies comprised five parallel-group trials and one two-period crossover trial. They evaluated onabotulinumtoxinA or incobotulinumtoxinA in adults with upper-limb essential tremor but differed in dose, muscle selection, injection guidance, treatment schedule, outcome instruments, and follow-up duration (Table 1).

**Table 1:** Characteristics of included randomized placebo-controlled studies of botulinum toxin type A for upper-limb essential tremor.

Study/source	Design, setting, and participants	BoNT-A regimen and targeting	Eligible outcomes/follow-up	Contribution to synthesis
Brin et al. (29)	Three-arm, double-masked parallel RCT; 10 centres (USA/Canada). *N* = 133 (placebo 45; onabotulinumtoxinA 50 U, 43; 100 U, 45). Mean age 68.5 years; 90 men/43 women; essential hand tremor (TRIG criteria).	OnabotulinumtoxinA 50 or 100 U; fixed wrist-flexor/extensor injections; one treatment; vehicle placebo.	Clinician-rated postural/kinetic tremor, investigator- and patient-rated global response, function, grip strength and adverse events; Weeks 1–16.	Contributed to tremor severity, global-response and grip-strength meta-analyses. Active arms were combined against the shared placebo group; alternative tremor and grip-strength measures were tested in sensitivity analyses.
Jankovic et al. (30)	Single-center, double-blind parallel RCT (USA). *N* = 25 (BoNT-A 13; placebo 12); rescue dosing allowed after Week 4. Typical disabling essential hand tremor.	OnabotulinumtoxinA 50 U into wrist flexors/extensors using anatomical landmarks; matched placebo.	Clinician-rated tremor, function, examiner/patient global ratings, accelerometry, hand force and weakness; randomized comparison at Week 4 before rescue dosing.	Contributed to investigator- and patient-rated global-response analyses. Responder data were used only in an exploratory sensitivity analysis; function and safety were summarized individually.
Mittal et al. (28)	Single-center, double-blind crossover RCT (USA); *N* = 33 randomized, 28 completed; crossover at Week 16; total follow-up 28 weeks. Moderate-to-severe essential hand tremor.	IncobotulinumtoxinA 80–120 U (about 100 U) into 8–14 hand/forearm muscles; individualized clinical and EMG-guided targeting; saline placebo.	FTM, NIHCGC tremor severity, PGIC, grip strength and muscular weakness after each treatment period.	Narrative synthesis only. No paired treatment effect with variance, paired binary table, sufficient participant-level paired data, or separable first-period results.
Jog et al. (31)	Multicentre, double-blind parallel RCT (Canada/USA); 2:1 allocation; *N* = 30 (incobotulinumtoxinA 19; placebo 11). Mean age 68.1 years; 15 women/15 men; definite upper-limb ET.	IncobotulinumtoxinA 30–200 U (mean 116.3 U); individualized kinematic plan with ultrasound, EMG and/or electrical-stimulation guidance; one treatment.	FTM outcomes, global response, kinematic tremor, grip strength, TEAEs, SAEs and discontinuations; follow-up through Week 24.	Contributed to tremor severity, global response, grip strength and all eligible 24-week safety analyses.
NCT04766723/EudraCT 2021-001988-24	Multicentre, double-blind placebo-controlled randomized Cycle 1; 14 sites (USA/Canada/Poland). *N* = 78 randomized (NT 201, 51; placebo, 27); adult motor-dominant upper-limb ET.	IncobotulinumtoxinA 130–165 U in the motor-dominant upper limb; semi-flexible muscle/dose approach; randomized unilateral Cycle 1 through Week 24.	Kinematic and TETRAS outcomes, investigator/participant global response, ADL/function, weakness, treatment-related TEAEs, SAEs and discontinuations.	Only traceable randomized Cycle-1 results contributed to meta-analyses. Outcome-specific denominators varied by randomized, treated and evaluable populations.
ELATE / NCT05216250	Phase 2 multicentre, double-blind parallel RCT (USA/Canada); two blinded treatment cycles. ClinicalTrials.gov reported an enrollment of 174 participants. Adults were aged 18–80 years and had upper-limb essential tremor. Detailed randomized-group demographics were not publicly reported.	OnabotulinumtoxinA versus placebo at baseline and Week 12; public sources did not provide complete muscle-level dose details.	TREDS-R, TETRAS ADL/upper-limb measures, clinician/patient outcomes and safety; repeated-treatment assessment at Week 18.	Narrative emerging evidence only because no complete peer-reviewed result report or fully traceable group-level dataset was available.

BoNT-A, botulinum toxin type A; ADL, activities of daily living; EMG, electromyography; ET, essential tremor; FTM, Fahn–Tolosa–Marín; NIHCGC, National Institutes of Health Collaborative Genetic Criteria; PGIC, Patient Global Impression of Change; SAE, serious adverse event; TEAE, treatment-emergent adverse event; TETRAS, The Essential Tremor Rating Assessment Scale; TREDS-R, Tremor Disability Scale-Revised; TRIG, Tremor Investigation Group. Mittal (2018) was included in the systematic review and narrative synthesis but not in any meta-analysis.

Mittal (2018) was retained as eligible crossover evidence but was synthesized narratively because suitable paired treatment effects, paired binary tables, and separable first-period data were unavailable (28). ELATE/NCT05216250 was also summarized narratively because the available registry-, sponsor-, and conference-level sources did not provide a complete and fully traceable peer-reviewed dataset (18–20).

Jankovic (1996) and Brin (2001) used relatively fixed injections into wrist flexor and extensor muscles (29, 30). Mittal (2018) used individualized clinical and electromyographic muscle selection (28), Jog (2020) used a kinematically guided individualized injection plan (31), and NCT04766723 used a semi-flexible approach to muscle selection and dosing (16, 17).

Brin (2001) randomized 133 participants to placebo or one of two onabotulinumtoxinA doses. For eligible quantitative outcomes, the two active groups were combined and compared once with the shared placebo group (29). Jankovic (1996) permitted rescue dosing after Week 4; only the randomized comparison before rescue dosing was used (30). Mittal (2018) randomized 33 participants in a crossover design, of whom 28 completed both treatment periods (28). Its treatment-period observations were not treated as independent parallel-group data. NCT04766723 randomized 78 participants; only results from randomized unilateral Cycle 1 were eligible for quantitative synthesis (16, 17). ELATE/NCT05216250 evaluated two blinded treatment cycles. ClinicalTrials.gov reported an enrollment of 174 participants (18).

### 3.3 Risk of bias

Risk of bias was assessed for each eligible result. Among 26 parallel-group result-level assessments, 2 (7.7%) were judged to be at low risk of bias, 19 (73.1%) raised some concerns, and 5 (19.2%) were judged to be at high overall risk (Supplementary Figures S1A,B).

Bias due to deviations from intended interventions was judged to be low for all parallel-group results. The most frequent limitations involved outcome measurement and selection of the reported result. Subjective outcomes from older trials were vulnerable to functional unblinding because recognizable weakness could reveal treatment allocation. Protocols and statistical analysis plans were unavailable for several trials, raising concerns regarding selective reporting.

The three assessed Mittal outcomes—Week-4 FTM score, muscular weakness, and PGIC—were each judged to be at high overall risk of bias. Principal concerns included missing outcome data, insufficient reporting of paired binary analyses, and selective reporting. The period and carryover domain raised some concerns because original treatment-sequence counts and numerical carryover-test results were incompletely reported, although the 16-week interval was probably sufficient for the treatment effect to diminish (28).

### 3.4 Primary efficacy outcome: short-term clinician-rated upper-limb tremor severity

**Figure 2:**
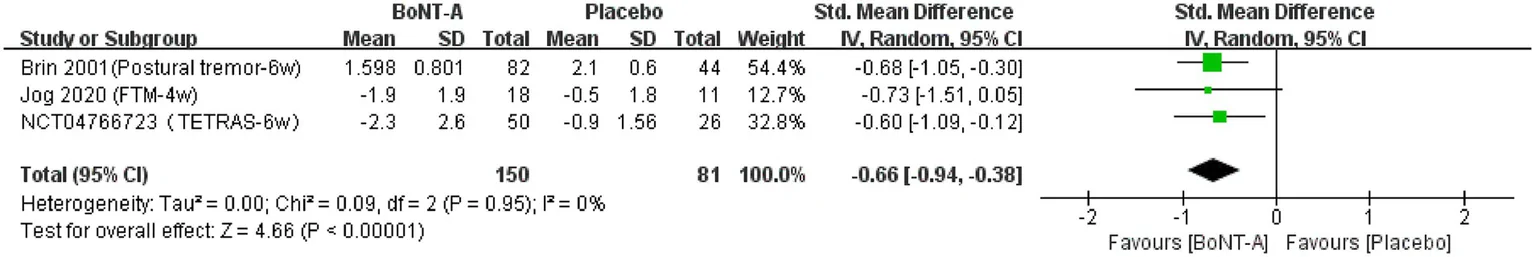
Forest plot of short-term clinician-rated upper-limb tremor severity. Standardized effects are expressed as Hedges’ *g* with 95% confidence intervals. Negative values favor BoNT-A.

Three parallel-group trials involving 231 participants contributed to the primary efficacy analysis (16, 17, 29, 31). At 4–6 weeks, BoNT-A reduced clinician-rated upper-limb tremor severity compared with placebo (Hedges’ g, −0.66; 95% CI, −0.94 to −0.38; I^2^ = 0%; Figure 2). Certainty of evidence was moderate. Sensitivity analyses produced similar estimates. Substitution of the Brin kinetic-tremor measure produced a pooled standardized mean difference of −0.70 (95% CI, −0.97 to −0.42; I^2^ = 0%). Exclusion of Brin 2001 produced Hedges’ g − 0.64 (95% CI, −1.05 to −0.23; I^2^ = 0%). An exploratory analysis adding the converted Jankovic responder result produced a pooled standardized mean difference of −0.70 (95% CI, −0.97 to −0.43; I^2^ = 0%). Results are summarized in Supplementary Table S4 and Supplementary Figures S2A–C.

### 3.5 Short-term global treatment response

#### 3.5.1 Investigator-rated global treatment response

**Figure 3:**
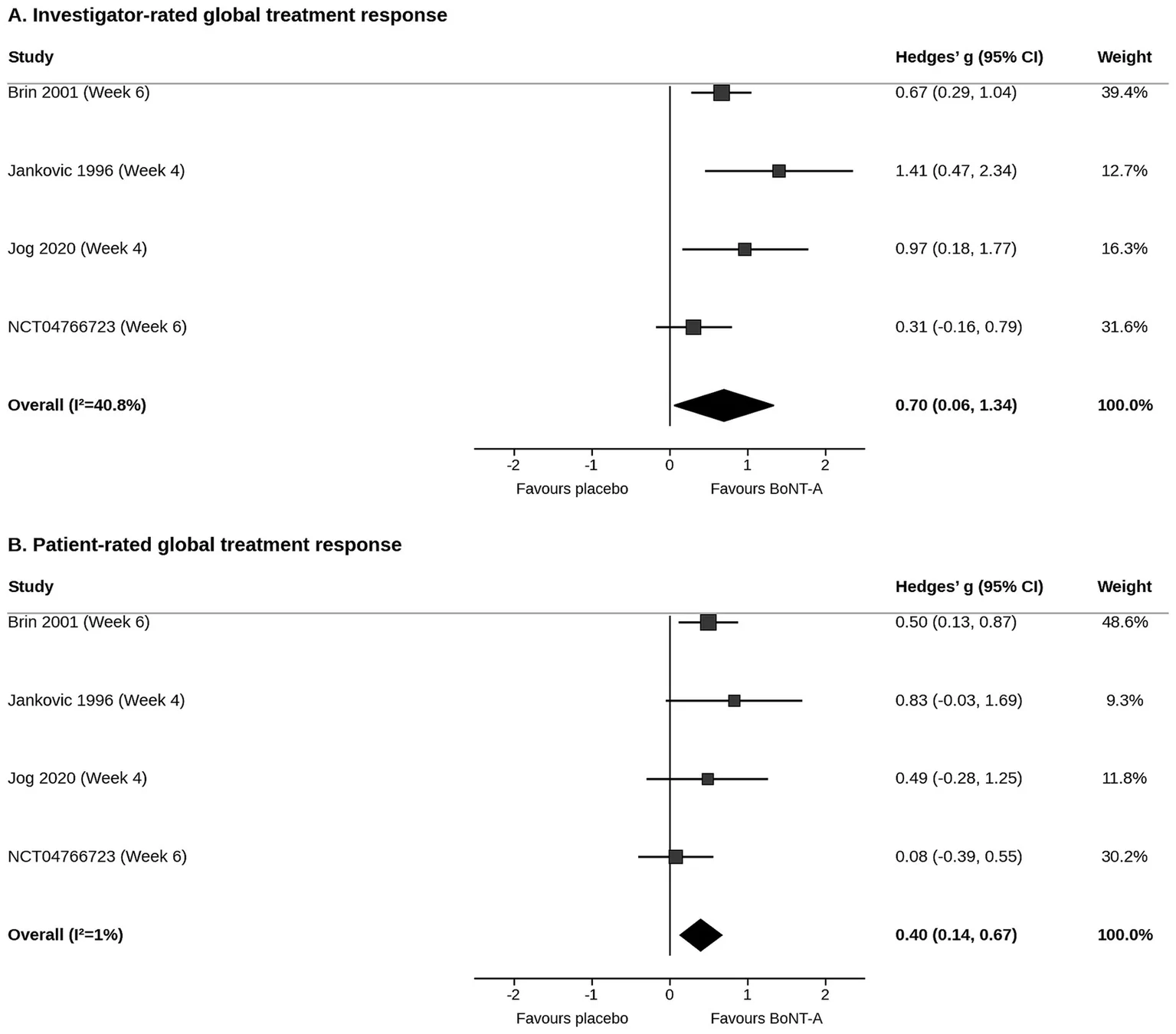
Forest plots of short-term global treatment response. **(A)** Investigator-rated global response, and **(B)** patient-rated global response. The two outcomes were analyzed separately because they were obtained from overlapping participants; no combined overall effect was calculated.

Four trials with outcome data from 254 participants contributed to this analysis (16, 17, 29–31). The pooled effect favored BoNT-A (Hedges’ g, 0.70; 95% CI, 0.06 to 1.34; I^2^ = 40.8%; low-certainty evidence; Figure 3A). Brin (2001) assessed response relative to the preceding visit, whereas the other studies assessed improvement relative to baseline or overall treatment status. After exclusion of Brin (2001), the point estimate remained favorable, but the modified Hartung–Knapp confidence interval crossed the null (Hedges’ g, 0.80; 95% CI, −0.63 to 2.23; I^2^ = 60.4%; Supplementary Figure S2D).

#### 3.5.2 Patient-rated global treatment response

Four trials with outcome data from 254 participants contributed to the patient-rated analysis (16, 17, 29–31). The pooled estimate favored BoNT-A (Hedges’ g, 0.40; 95% CI, 0.14 to 0.67; I^2^ = 1%; Figure 3B). Certainty was low. After exclusion of Brin (2001) and application of modified Hartung–Knapp inference, the point estimate remained positive but the confidence interval crossed the null (Hedges’ g, 0.36; 95% CI, −0.63 to 1.34; I^2^ = 21.1%; Supplementary Figure S2E).

Investigator- and patient-rated outcomes were analyzed separately because they were obtained from overlapping participants. No common overall estimate or subgroup-difference test between raters was calculated.

### 3.6 Grip strength, function, activities of daily living, and tremor-related disability

**Figure 4:**
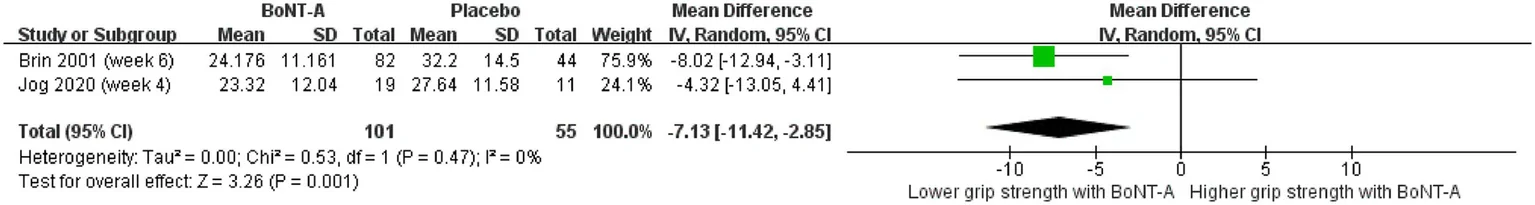
Forest plot of maximum grip strength at 4–6 weeks. Effects are expressed as mean differences in kilograms with 95% confidence intervals. Negative values indicate lower grip strength with BoNT-A.

Two trials involving 156 participants reported maximum grip strength at 4–6 weeks (29, 31). BoNT-A was associated with lower grip strength than placebo (mean difference, −7.13 kg; 95% CI, −11.42 to −2.85; I^2^ = 0%; low-certainty evidence; Figure 4). Substitution of the alternative Brin measurement produced a similar result (mean difference, −7.68 kg; 95% CI, −12.00 to −3.35; I^2^ = 0%; Supplementary Figure S2F).

Functional and activities-of-daily-living outcomes were not pooled because the instruments and estimands differed. Jankovic (1996) showed no clear between-group difference in the UTRA functional score (30). Jog (2020) reported an adjusted Week-4 FTM Part B effect favoring BoNT-A (mean difference, −2.30; SE, 0.74) (31). In NCT04766723, changes in upper-limb TETRAS ADL and functional-impact measures were similar between groups (16, 17). Individual results are presented in Supplementary Table S4.

Device-measured tremor results were also reported separately because devices, units, and statistical models differed. Jog (2020) reported an adjusted Week-4 reduction in log-transformed accelerometric tremor amplitude (least-squares mean difference, −0.66; 95% CI, −1.09 to −0.23) (31).

### 3.7 Safety outcomes

#### 3.7.1 Primary safety outcome: muscular weakness

**Figure 5:**
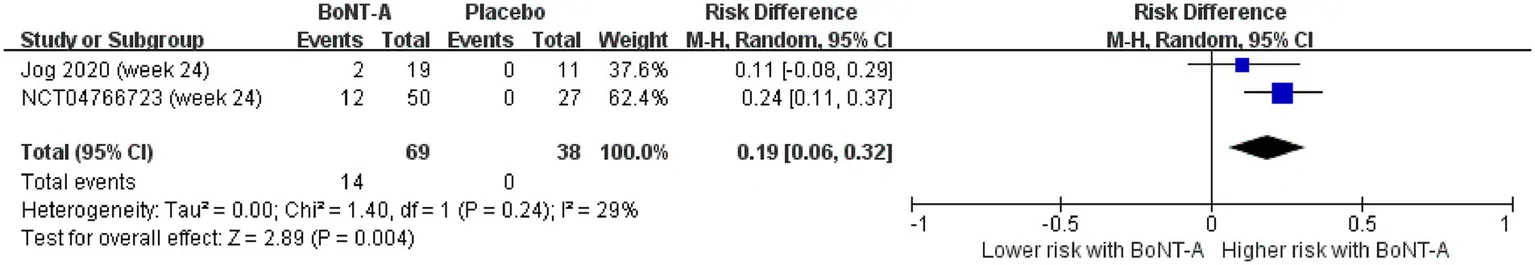
Forest plot of muscular weakness during randomized follow-up through Week 24. Effects are expressed as risk differences with 95% confidence intervals. Positive values indicate greater risk with BoNT-A.

Two trials involving 107 participants contributed to the primary muscular-weakness analysis through Week 24 (16, 17, 31). Weakness occurred in 14 of 69 BoNT-A participants and 0 of 38 placebo participants. The pooled risk difference was 0.19 (95% CI, 0.06 to 0.32; I^2^ = 29%; Figure 5), equivalent to approximately 190 more participants experiencing muscular weakness per 1,000 treated (95% CI, 60 to 320 more per 1,000). Certainty was low.

Weakness at other follow-up windows was not combined with the 24-week estimate. Jankovic (1996) reported mild or moderate finger weakness in 11 of 12 BoNT-A participants and 0 of 11 placebo participants at Week 4 (30). Brin (2001) reported hand weakness in 44 of 88 BoNT-A participants through Week 16 but did not report a corresponding placebo-group event count; therefore, no comparative effect was calculated (29).

#### 3.7.2 Treatment-related adverse events

Two trials involving 107 participants reported participants with at least one treatment-related treatment-emergent adverse event through Week 24 (16, 17, 31). Events occurred in 18 of 69 BoNT-A participants and 2 of 38 placebo participants. The pooled risk difference was 0.19 (95% CI, 0.00 to 0.37; I^2^ = 48%; Supplementary Figure S3A). Certainty was very low, and the estimate was compatible with both no difference and an important increase in risk.

#### 3.7.3 Serious adverse events

Serious adverse events occurred in 3 of 69 BoNT-A participants and 4 of 38 placebo participants across two trials (16, 17, 31). The pooled risk difference was −0.07 (95% CI, −0.22 to 0.09; I^2^ = 37%; low-certainty evidence; Supplementary Figure S3B). The estimate was too imprecise to establish benefit or harm.

#### 3.7.4 Discontinuation due to adverse events

Discontinuation due to adverse events occurred in 1 of 70 BoNT-A participants and 1 of 38 placebo participants across two trials (16, 17, 31). The pooled risk difference was −0.01 (95% CI, −0.11 to 0.10; I^2^ = 31%; Supplementary Figure S3C). Certainty was low, and the sparse events precluded a reliable conclusion.

### 3.8 Eligible studies not included in the meta-analyses

Mittal (2018) was an eligible randomized crossover trial involving 33 participants, of whom 28 completed both periods (28). At Week 4, the paired FTM comparison favored incobotulinumtoxinA (Wilcoxon signed-rank *p* < 0.003). However, no paired treatment-effect estimate with corresponding variance, sufficient participant-level paired data, or separable first-period results was reported. PGIC data were available for 19 participants; 10 reported being “much improved” during the incobotulinumtoxinA period and 3 during the placebo period (*p* = 0.031). Mild weakness was reported during 6 incobotulinumtoxinA periods and 4 placebo periods, and one participant withdrew because of unacceptable weakness. Paired discordant-cell data were unavailable; therefore, no binary comparative effect was calculated.

ELATE/NCT05216250 evaluated two blinded treatment cycles (18–20). Publicly available sponsor-reported topline results indicated least-squares mean TREDS-R changes of −2.61 with onabotulinumtoxinA and −1.61 with placebo (*p* = 0.029). Muscular weakness was reported in 24.5 and 2.3% of participants, respectively (20). These findings were not pooled because a complete peer-reviewed report, reconciled analysis populations, and fully traceable group-level data were unavailable. These narrative-only results are also summarized in Supplementary Table S4.

### 3.9 Certainty of evidence

The complete GRADE Evidence Profile is presented in Supplementary Table S5, and the seven outcomes considered most important for clinical decision-making are summarized in Table 2.

**Table 2:** Summary of findings: Botulinum toxin type A versus placebo for upper-limb essential tremor.

Outcome/follow-up	Placebo risk or reference	BoNT-A absolute risk / effect	Difference or pooled effect (95% CI)	Participants (studies)	Certainty	Importance	Plain-language interpretation	Footnote
Clinician-rated upper-limb tremor severity; 4–6 weeks	Reference distribution	0.66 SD lower	SMD 0.66 lower (0.94 lower to 0.38 lower)	231 (3 RCTs)	Moderate ⊕ ⊕ ⊕◯	Critical	BoNT-A probably reduces short-term clinician-rated tremor severity; clinical importance remains uncertain because no validated cross-scale MID was available.	a
Muscular weakness; through Week 24	0 per 1,000	190 per 1,000 (60–320)	190 more per 1,000 (60 more to 320 more)	107 (2 RCTs)	Low ⊕ ⊕ ◯◯	Critical	BoNT-A may increase muscular weakness.	b
Patient-rated global treatment response; 4–6 weeks	Reference distribution	0.40 SD higher	SMD 0.40 higher (0.14 higher to 0.67 higher)	254 (4 RCTs)	Low ⊕ ⊕ ◯◯	Critical	BoNT-A may improve patient-rated global response, but the magnitude and robustness are uncertain.	c
Maximum grip strength; 4–6 weeks	Mean grip strength in placebo groups	7.13 kg lower	MD 7.13 kg lower (11.42 lower to 2.85 lower)	156 (2 RCTs)	Low ⊕ ⊕ ◯◯	Important	BoNT-A may reduce short-term maximum grip strength.	d
Treatment-related TEAEs; through Week 24	53 per 1,000	243 per 1,000 (53–423)	190 more per 1,000 (0 to 370 more)	107 (2 RCTs)	Very low ⊕◯◯◯	Critical	The evidence is very uncertain about treatment-related adverse events.	e
Serious adverse events; through Week 24	105 per 1,000	35 per 1,000 (0–195)	70 fewer per 1,000 (220 fewer to 90 more)	107 (2 RCTs)	Low ⊕ ⊕ ◯◯	Critical	BoNT-A may have little or no effect; important benefit or harm cannot be excluded.	f
Discontinuation due to adverse events; through Week 24	26 per 1,000	16 per 1,000 (0–126)	10 fewer per 1,000 (110 fewer to 100 more)	108 (2 RCTs)	Low ⊕ ⊕ ◯◯	Important	BoNT-A may have little or no effect; important benefit or harm cannot be excluded.	g

Population: Adults with upper-limb essential tremor | Setting: Outpatient/specialist care | Intervention: BoNT-A injection | Comparator: Placebo or saline. GRADE certainty: High ⊕⊕⊕⊕; Moderate ⊕⊕⊕◯; Low ⊕⊕◯◯; Very low ⊕◯◯◯. Absolute effects are derived from the pooled risk difference and aggregate placebo risk. SMD, standardized mean difference; MD, mean difference; MID, minimal important difference; RCT, randomized controlled trial; TEAE, treatment-emergent adverse event. Footnotes a–g are provided in Supplementary Table S5.

Certainty was moderate for short-term clinician-rated tremor severity. Certainty was low for muscular weakness, investigator-rated global response, patient-rated global response, maximum grip strength, serious adverse events, and discontinuation due to adverse events. Certainty was very low for treatment-related treatment-emergent adverse events. No outcome was supported by high-certainty evidence.

The principal reasons for downgrading were risk of bias and imprecision arising from small numbers of studies, participants, and events. Mittal (2018) was considered as additional narrative evidence but did not increase the study or participant count for any pooled GRADE estimate.

## 4 Discussion

This review found moderate-certainty evidence that BoNT-A probably reduces short-term clinician-rated upper-limb tremor severity. Evidence for investigator- and patient-rated global treatment response was of low certainty and was sensitive to exclusion of Brin (2001). BoNT-A was also associated with reduced grip strength and increased muscular weakness. Evidence regarding broader and less frequent adverse events remained limited or very uncertain. Overall, the findings indicate a trade-off between short-term tremor reduction and treatment-related weakness rather than a uniformly favorable benefit–harm profile.

The primary efficacy estimate remained directionally consistent across alternative outcome selections. However, its clinical importance remains uncertain because different clinician-rated scales were combined and no validated cross-scale minimal important difference was available. Improvement in tremor ratings also did not translate consistently into better function. The decrease in grip strength and mixed functional and ADL findings illustrate the biological trade-off of peripheral chemodenervation: weakening tremor-generating muscles may reduce oscillation while impairing force and task performance (9, 10, 29–31).

Investigator- and patient-rated responses were analyzed separately to avoid treating overlapping observations as independent. Although both primary estimates favored BoNT-A, exclusion of Brin (2001) produced wide modified Hartung–Knapp intervals that crossed the null. This sensitivity reflects differences in the reference frame of global-response measures and the small number of studies. Study-specific binary responder outcomes were interpreted separately because their definitions, thresholds, raters, assessment times, and statistical structures differed.

Muscular weakness was the most consistent treatment-related harm. The pooled 24-week estimate indicated an absolute increase of approximately 19 percentage points, while earlier fixed-injection trials also reported frequent weakness (29, 30). By contrast, treatment-related adverse events, serious adverse events, and discontinuations were based on few events and wide confidence intervals. These results neither establish an acceptable overall safety profile nor demonstrate clear excess serious harm; they indicate that weakness and possible functional impairment should be discussed explicitly when treatment is considered.

Injection strategies varied from fixed wrist-flexor and extensor patterns to individualized EMG-, ultrasound-, electrical-stimulation-, or kinematic-guided approaches (16, 17, 28–32). Toxin formulation, total dose, dose per muscle, number of injected muscles, laterality, and retreatment schedules also differed. These factors may influence both efficacy and weakness, but injection strategy was confounded with study period, formulation, dose, outcome measurement, follow-up, and risk of bias. Current randomized evidence therefore does not establish that customized or device-guided injection is superior to fixed injection, although individualized targeting remains clinically plausible.

Mittal (2018) and ELATE provided relevant additional evidence but did not contribute to pooled estimates because suitable paired data or complete, traceable group-level results were unavailable. The Mittal outcomes were also judged to be at high overall risk of bias (28), and ELATE remained available primarily through registry and conference-level sources; their findings should therefore be interpreted as supportive but uncertain evidence (18–20).

Previous reviews have reached differing conclusions. Zheng et al. reported no clear overall benefit for hand tremor and an increase in adverse events, whereas Liao et al. reported short-term tremor improvement (12, 13). The 2019 and 2026 MDS evidence-based reviews remained cautious because of small trials, heterogeneous methods, and limited follow-up (7, 8). By restricting eligibility to upper-limb essential tremor, the present review avoids combining distinct anatomical treatment targets and diagnostic populations. Its findings should not be extrapolated to dystonic, functional, parkinsonian, orthostatic, or isolated head tremor (1, 33).

The evidence does not establish the optimal position of BoNT-A in the treatment sequence for essential tremor. Current guidelines and evidence reviews support cautious individualized use rather than a categorical recommendation (6–8, 11). BoNT-A may be considered as a targeted option for selected adults with upper-limb essential tremor, but the evidence does not demonstrate that it should be used routinely, that it is restricted to medically refractory disease, or that a particular clinical subgroup derives greater benefit. Decisions should consider tremor distribution, patient-prioritized functional goals, baseline strength, comorbidities, previous treatment experience, and expertise in muscle selection and dosing.

Strengths of this review include the expanded multilingual and grey-literature search, linkage of multiple reports from the same trial, source-level verification of quantitative data, appropriate handling of shared placebo groups and crossover trials, result-level RoB 2 assessment, and outcome-specific GRADE evaluation. Important limitations remain. The review was not prospectively registered, and several eligibility and analytical decisions were revised during peer review; consequently, *post hoc* analytical flexibility cannot be excluded. Each synthesis included only two to four studies, many outcomes involved small samples or sparse events, and confidence intervals were often wide. Clinical diversity in diagnostic criteria, formulation, dose, muscle selection, guidance, outcome measurement, and follow-up limited comparability. Several older trials lacked accessible protocols or statistical analysis plans, repeated-treatment evidence was incomplete, and no validated cross-scale minimal important difference was available. Publication bias could not be assessed reliably and cannot be excluded.

Future multicenter trials should be prospectively registered and should report protocols, statistical analysis plans, muscle-level dosing, injection guidance, repeated-treatment outcomes, standardized weakness grading, patient-prioritized function, quality of life, and complete adverse-event data. Crossover trials should additionally report paired effects, within-participant variances, period and carryover analyses, and paired binary tables.

## 5 Conclusion

In adults with upper-limb essential tremor, BoNT-A probably reduces short-term clinician-rated tremor severity, although the clinical importance of the standardized effect remains uncertain. Evidence for investigator- and patient-rated global response is of low certainty and is sensitive to analytical assumptions. Treatment is also associated with reduced grip strength and increased muscular weakness, while evidence regarding less frequent adverse outcomes remains limited or very uncertain.

BoNT-A may therefore be considered as an individualized, targeted option when the expected reduction in tremor outweighs the risk of weakness and functional impairment. The optimal dose, muscle-selection strategy, injection-guidance method, position in the treatment sequence, durability of benefit, and long-term benefit–harm balance remain uncertain. Larger prospectively registered trials with standardized patient-important outcomes and repeated-treatment follow-up are required.

## Data Availability

The original contributions presented in the study are included in the article/Supplementary material, further inquiries can be directed to the corresponding author.

## Supplementary material

SUPPLEMENTARY FIGURE S1 Result-level risk-of-bias assessment. **(A)** RoB 2 traffic-light plot for eligible parallel-group and crossover-trial results. The crossover-trial assessment includes the additional domain concerning period and carryover effects. **(B)** Distribution of RoB 2 judgments across the 26 parallel-group result-level assessments. Percentages are unweighted and do not include the Mittal 2018 crossover-trial results.

SUPPLEMENTARY FIGURE S2 Sensitivity analyses. **(A–C)** Show the three clinician-rated tremor-severity sensitivity analyses; **(D,E)** show investigator- and patient-rated global-response analyses excluding Brin (2001); **(F)** shows the alternative Brin grip-strength measurement.

SUPPLEMENTARY FIGURE S3 Secondary safety outcomes through Week 24. **(A)** Shows treatment-related treatment-emergent adverse events; **(B)** shows serious adverse events; **(C)** shows discontinuation due to adverse events.
