# Supplementary material for: Efficacy and safety of botulinum toxin type a for upper-limb essential tremor: a systematic review and meta-analysis of randomized placebo-controlled trials
Source: Front Neurol. 2026 Aug 28;17:1886329. doi: 10.3389/fneur.2026.1886329 (PMC13561811; doi:10.3389/fneur.2026.1886329)
Supplement: Supplementary file 1 [file Table_1.DOCX]

**Supplementary Table S1. Complete search strategies, search dates and records retrieved**

Final search date: 20 July 2026 (Ovid MEDLINE executed on 17 July 2026). Searches were run from database inception without date restrictions. The electronic search was intentionally broader than the final upper-limb eligibility criteria to identify potentially relevant randomized botulinum-toxin evidence for eligibility reassessment. MEDLINE was searched through both PubMed and Ovid; overlap was removed during deduplication.

| **Source** | **Platform / interface** | **Date searched** | **Records** |
| --- | --- | --- | --- |
| **MEDLINE** | PubMed | 20 Jul 2026 | 114 |
| **Embase** | Embase.com | 20 Jul 2026 | 591 |
| **CENTRAL** | Cochrane Library | 20 Jul 2026 | 41 |
| **MEDLINE** | Ovid MEDLINE (1946–present) | 17 Jul 2026 | 188 |
| **ProQuest Dissertations & Theses Global** | ProQuest | 20 Jul 2026 | 111 |
| **Web of Science Core Collection** | Clarivate | 20 Jul 2026 | 220 |
| **CNKI** | China National Knowledge Infrastructure | 20 Jul 2026 | 0 |
| **WanFang Data** | WanFang | 20 Jul 2026 | 46 |
| **SinoMed** | Chinese Biomedical Literature Service System | 20 Jul 2026 | 54 |
| **ClinicalTrials.gov** | U.S. National Library of Medicine | 20 Jul 2026 | 9 |
| **WHO ICTRP** | WHO International Clinical Trials Registry Platform | 20 Jul 2026 | 9 |

Bibliographic databases: 1,365 records; trial registers: 18 records; total from databases and trial registers: 1,383 records.

**1. MEDLINE via PubMed**

(("Essential Tremor"[MeSH Terms] OR "essential tremor"[Title/Abstract] OR "essential tremors"[Title/Abstract] OR "benign essential tremor"[Title/Abstract] OR "benign essential tremors"[Title/Abstract] OR "familial tremor"[Title/Abstract] OR "familial tremors"[Title/Abstract] OR "hereditary essential tremor"[Title/Abstract] OR "hereditary tremor"[Title/Abstract] OR "senile tremor"[Title/Abstract] OR "essential hand tremor"[Title/Abstract] OR "essential head tremor"[Title/Abstract] OR "isolated head tremor"[Title/Abstract] OR "upper limb essential tremor"[Title/Abstract] OR "upper-limb essential tremor"[Title/Abstract] OR "essential voice tremor"[Title/Abstract] OR (("head tremor"[Title/Abstract] OR "hand tremor"[Title/Abstract] OR "upper limb tremor"[Title/Abstract] OR "upper-limb tremor"[Title/Abstract] OR "voice tremor"[Title/Abstract]) AND (essential[Title/Abstract] OR idiopathic[Title/Abstract] OR familial[Title/Abstract]))) AND ("Botulinum Toxins"[MeSH Terms] OR "Botulinum Toxins, Type A"[MeSH Terms] OR "botulinum toxin"[Title/Abstract] OR "botulinum toxins"[Title/Abstract] OR "botulinum neurotoxin"[Title/Abstract] OR "botulinum neurotoxins"[Title/Abstract] OR "botulinum toxin type A"[Title/Abstract] OR "botulinum toxin A"[Title/Abstract] OR "botulinum neurotoxin type A"[Title/Abstract] OR "botulinum neurotoxin A"[Title/Abstract] OR "botulinum A toxin"[Title/Abstract] OR "Clostridium botulinum toxin"[Title/Abstract] OR "Clostridium botulinum toxin type A"[Title/Abstract] OR "BoNT-A"[Title/Abstract] OR "BoNT/A"[Title/Abstract] OR "BTX-A"[Title/Abstract] OR onabotulinumtoxinA[Title/Abstract] OR incobotulinumtoxinA[Title/Abstract] OR abobotulinumtoxinA[Title/Abstract] OR prabotulinumtoxinA[Title/Abstract] OR daxibotulinumtoxinA[Title/Abstract] OR letibotulinumtoxinA[Title/Abstract] OR Botox[Title/Abstract] OR Dysport[Title/Abstract] OR Xeomin[Title/Abstract] OR Jeuveau[Title/Abstract] OR Nuceiva[Title/Abstract] OR Daxxify[Title/Abstract] OR Letybo[Title/Abstract] OR Meditoxin[Title/Abstract] OR Neuronox[Title/Abstract] OR Oculinum[Title/Abstract] OR Vistabel[Title/Abstract] OR Vistabex[Title/Abstract] OR Prosigne[Title/Abstract] OR Hengli[Title/Abstract]) AND (randomized controlled trial[Publication Type] OR controlled clinical trial[Publication Type] OR randomized[Title/Abstract] OR randomised[Title/Abstract] OR placebo[Title/Abstract] OR drug therapy[MeSH Subheading] OR randomly[Title/Abstract] OR trial[Title/Abstract] OR groups[Title/Abstract])) NOT (animals[MeSH Terms] NOT humans[MeSH Terms])

**2. Embase via Embase.com**

('essential tremor'/exp OR 'essential tremor*':ti,ab,kw OR 'benign essential tremor*':ti,ab,kw OR 'familial tremor*':ti,ab,kw OR 'hereditary essential tremor*':ti,ab,kw OR 'hereditary tremor*':ti,ab,kw OR 'senile tremor*':ti,ab,kw OR 'essential hand tremor*':ti,ab,kw OR 'essential head tremor*':ti,ab,kw OR 'isolated head tremor*':ti,ab,kw OR 'upper limb essential tremor*':ti,ab,kw OR 'upper-limb essential tremor*':ti,ab,kw OR 'essential voice tremor*':ti,ab,kw OR (((head OR hand OR 'upper limb' OR voice) NEAR/2 tremor*):ti,ab,kw AND (essential OR idiopathic OR familial):ti,ab,kw)) AND ('botulinum toxin'/exp OR 'botulinum toxin a'/exp OR 'botulinum toxin*':ti,ab,kw OR 'botulinum neurotoxin*':ti,ab,kw OR 'botulinum toxin type a':ti,ab,kw OR 'botulinum toxin a':ti,ab,kw OR 'botulinum neurotoxin type a':ti,ab,kw OR 'botulinum neurotoxin a':ti,ab,kw OR 'botulinum a toxin':ti,ab,kw OR 'clostridium botulinum toxin*':ti,ab,kw OR 'bont-a':ti,ab,kw OR 'bont/a':ti,ab,kw OR 'btx-a':ti,ab,kw OR onabotulinumtoxina:ti,ab,kw OR incobotulinumtoxina:ti,ab,kw OR abobotulinumtoxina:ti,ab,kw OR prabotulinumtoxina:ti,ab,kw OR daxibotulinumtoxina:ti,ab,kw OR letibotulinumtoxina:ti,ab,kw OR botox:ti,ab,kw OR dysport:ti,ab,kw OR xeomin:ti,ab,kw OR jeuveau:ti,ab,kw OR nuceiva:ti,ab,kw OR daxxify:ti,ab,kw OR letybo:ti,ab,kw OR meditoxin:ti,ab,kw OR neuronox:ti,ab,kw OR oculinum:ti,ab,kw OR vistabel:ti,ab,kw OR vistabex:ti,ab,kw OR prosigne:ti,ab,kw OR hengli:ti,ab,kw)

**3. Cochrane Central Register of Controlled Trials (CENTRAL) via the Cochrane Library**

#1 MeSH descriptor: [Essential Tremor] explode all trees
#2 ("essential tremor" OR "essential tremors" OR "benign essential tremor" OR "familial tremor" OR "hereditary essential tremor" OR "hereditary tremor" OR "senile tremor" OR "essential hand tremor" OR "essential head tremor" OR "isolated head tremor" OR "upper limb essential tremor" OR "upper-limb essential tremor" OR "essential voice tremor"):ti,ab,kw
#3 (((head OR hand OR "upper limb" OR voice) NEAR/2 tremor*) AND (essential OR idiopathic OR familial)):ti,ab,kw
#4 #1 OR #2 OR #3
#5 MeSH descriptor: [Botulinum Toxins] explode all trees
#6 MeSH descriptor: [Botulinum Toxins, Type A] explode all trees
#7 ("botulinum toxin" OR "botulinum toxins" OR "botulinum neurotoxin" OR "botulinum neurotoxins" OR "botulinum toxin type A" OR "botulinum toxin A" OR "botulinum neurotoxin type A" OR "botulinum neurotoxin A" OR "botulinum A toxin" OR "Clostridium botulinum toxin" OR BoNT-A OR "BoNT/A" OR BTX-A OR onabotulinumtoxinA OR incobotulinumtoxinA OR abobotulinumtoxinA OR prabotulinumtoxinA OR daxibotulinumtoxinA OR letibotulinumtoxinA OR Botox OR Dysport OR Xeomin OR Jeuveau OR Nuceiva OR Daxxify OR Letybo OR Meditoxin OR Neuronox OR Oculinum OR Vistabel OR Vistabex OR Prosigne OR Hengli):ti,ab,kw
#8 #5 OR #6 OR #7
#9 #4 AND #8 [41 records]

**4. MEDLINE via Ovid (1946 to 17 July 2026)**

1. exp Essential Tremor/
2. (essential adj2 tremor*).ti,ab,kf.
3. (benign essential tremor* or familial tremor* or hereditary essential tremor* or hereditary tremor* or senile tremor* or essential hand tremor* or essential head tremor* or isolated head tremor* or upper limb essential tremor* or upper-limb essential tremor* or essential voice tremor*).ti,ab,kf.
4. (((essential or idiopathic or familial) adj3 (head tremor* or hand tremor* or upper limb tremor* or upper-limb tremor* or voice tremor*)) or isolated head tremor* or isolated voice tremor*).ti,ab,kf.
5. or/1-4
6. exp Botulinum Toxins/
7. exp Botulinum Toxins, Type A/
8. (botulinum adj2 toxin*).ti,ab,kf.
9. (botulinum adj2 neurotoxin*).ti,ab,kf.
10. (botulinum toxin type A or botulinum toxin A or botulinum neurotoxin type A or botulinum neurotoxin A or botulinum A toxin or Clostridium botulinum toxin or Clostridium botulinum toxin type A).ti,ab,kf.
11. ((BoNT adj1 A) or (BTX adj1 A) or "BoNT/A" or BoNTA or BTXA).ti,ab,kf.
12. (onabotulinumtoxinA* or incobotulinumtoxinA* or abobotulinumtoxinA* or prabotulinumtoxinA* or daxibotulinumtoxinA* or letibotulinumtoxinA*).ti,ab,kf.
13. (Botox or Dysport or Xeomin or Jeuveau or Nuceiva or Daxxify or Letybo or Meditoxin or Neuronox or Oculinum or Vistabel or Vistabex or Prosigne or Hengli).ti,ab,kf.
14. or/6-13
15. randomized controlled trial.pt.
16. controlled clinical trial.pt.
17. randomized.ab.
18. randomised.ab.
19. placebo.ab.
20. randomly.ab.
21. trial.ab.
22. groups.ab.
23. or/15-22
24. animals/ not humans/
25. 5 and 14 and 23
26. 25 not 24 [188 records]

**5. ProQuest Dissertations & Theses Global**

NOFT("essential tremor" OR "essential tremors" OR "benign essential tremor" OR "familial tremor" OR "hereditary essential tremor" OR "hereditary tremor" OR "essential hand tremor" OR "essential head tremor" OR "isolated head tremor" OR "upper limb essential tremor" OR "upper-limb essential tremor" OR "essential voice tremor") AND NOFT("botulinum toxin" OR "botulinum toxins" OR "botulinum neurotoxin" OR "botulinum toxin type A" OR "botulinum toxin A" OR "botulinum neurotoxin type A" OR BoNT-A OR "BoNT/A" OR BTX-A OR onabotulinumtoxinA OR incobotulinumtoxinA OR abobotulinumtoxinA OR Botox OR Dysport OR Xeomin)

**6. Web of Science Core Collection**

TS=("essential tremor*" OR "benign essential tremor*" OR "familial tremor*" OR "hereditary essential tremor*" OR "hereditary tremor*" OR "senile tremor*" OR "essential hand tremor*" OR "essential head tremor*" OR "isolated head tremor*" OR "upper limb essential tremor*" OR "upper-limb essential tremor*" OR "essential voice tremor*") AND TS=("botulinum toxin*" OR "botulinum neurotoxin*" OR "botulinum toxin type A" OR "botulinum toxin A" OR "botulinum neurotoxin type A" OR "botulinum neurotoxin A" OR BoNT-A OR "BoNT/A" OR BTX-A OR onabotulinumtoxinA OR incobotulinumtoxinA OR abobotulinumtoxinA OR prabotulinumtoxinA OR daxibotulinumtoxinA OR letibotulinumtoxinA OR Botox OR Dysport OR Xeomin OR Jeuveau OR Nuceiva OR Daxxify OR Letybo OR Meditoxin OR Neuronox OR Oculinum OR Vistabel OR Vistabex OR Prosigne OR Hengli)

**7. China National Knowledge Infrastructure (CNKI)**

主题 = (特发性震颤 OR 原发性震颤 OR 良性特发性震颤 OR 家族性震颤 OR 遗传性震颤 OR 特发性手震颤 OR 原发性手震颤 OR 特发性头部震颤 OR 原发性头部震颤 OR 孤立性头部震颤 OR 上肢特发性震颤 OR 特发性声音震颤) AND 主题 = (肉毒毒素 OR 肉毒杆菌毒素 OR 肉毒神经毒素 OR A型肉毒毒素 OR A型肉毒杆菌毒素 OR A型肉毒神经毒素 OR 肉毒毒素A型 OR 保妥适 OR 衡力 OR 吉适 OR Botox OR Dysport OR Xeomin OR onabotulinumtoxinA OR incobotulinumtoxinA OR abobotulinumtoxinA) [0 records]

**8. WanFang Data**

(主题=(特发性震颤 OR 原发性震颤 OR 良性特发性震颤 OR 家族性震颤 OR 遗传性震颤 OR 特发性手震颤 OR 原发性手震颤 OR 特发性头部震颤 OR 原发性头部震颤 OR 孤立性头部震颤 OR 上肢特发性震颤 OR 特发性声音震颤)) AND 主题=(肉毒毒素 OR 肉毒杆菌毒素 OR 肉毒神经毒素 OR A型肉毒毒素 OR A型肉毒杆菌毒素 OR A型肉毒神经毒素 OR 肉毒毒素A型 OR 保妥适 OR 衡力 OR 吉适 OR Botox OR Dysport OR Xeomin) [46 records]

**9. SinoMed**

("特发性震颤"[常用字段] OR "原发性震颤"[常用字段] OR "良性特发性震颤"[常用字段] OR "家族性震颤"[常用字段] OR "遗传性震颤"[常用字段] OR "特发性手震颤"[常用字段] OR "特发性头部震颤"[常用字段] OR "原发性头部震颤"[常用字段] OR "孤立性头部震颤"[常用字段] OR "上肢特发性震颤"[常用字段]) AND ("肉毒毒素"[常用字段] OR "肉毒杆菌毒素"[常用字段] OR "肉毒神经毒素"[常用字段] OR "A型肉毒毒素"[常用字段] OR "A型肉毒杆菌毒素"[常用字段] OR "A型肉毒神经毒素"[常用字段] OR "保妥适"[常用字段] OR "衡力"[常用字段] OR "吉适"[常用字段]) [54 records]

**10. ClinicalTrials.gov**

```
Condition or disease: ("essential tremor" OR "essential head tremor" OR "isolated head tremor" OR "upper limb essential tremor")
Other terms: ("botulinum toxin" OR "botulinum neurotoxin" OR Botox OR onabotulinumtoxinA OR incobotulinumtoxinA OR abobotulinumtoxinA OR prabotulinumtoxinA OR daxibotulinumtoxinA)  [9 records]
```

**11. WHO International Clinical Trials Registry Platform (ICTRP)**

("essential tremor" OR "essential head tremor" OR "isolated head tremor" OR "upper limb essential tremor") AND ("botulinum toxin" OR "botulinum neurotoxin" OR Botox OR onabotulinumtoxinA OR incobotulinumtoxinA OR abobotulinumtoxinA OR prabotulinumtoxinA OR daxibotulinumtoxinA) [9 records]

**12. Supplementary search methods**

**12.1** Backward reference checking of included reports and relevant systematic reviews, including the 2020 and 2022 meta-analyses and later evidence reviews.

**12.2** Forward citation searching of eligible and included upper-limb placebo-controlled trial reports using Web of Science Core Collection and supplementary citation-search tools available to the review team.

**12.3** Checking trial registries, sponsor webpages, protocols, statistical analysis plans, results postings, and regulatory or conference-level sources for completed trials without a full peer-reviewed publication.

**12.4** Linking journal articles, conference abstracts, registry entries, protocols, statistical analysis plans, sponsor reports and result records arising from the same underlying study before counting studies.

**12.5** Publicly available sources were exhausted for crossover and registry-only evidence. Mittal 2018 was retained for narrative synthesis because no paired effect estimate, paired variance, paired binary table, or separable first-period data were available.

**Search-count summary used for the updated PRISMA 2020 flow diagram**

| **Item** | **Count** |
| --- | --- |
| **Records identified from bibliographic databases and trial registers** | 1,383 |
| **Records identified through corrected forward citation searching** | 405 |
| **Total records identified** | 1,788 |
| **Duplicate records removed** | 901 (668 database/register; 233 forward citation) |
| **Unique records screened** | 887 |
| **Studies included in the systematic review** | 6 |
| **Studies contributing to at least one meta-analysis** | 4 |
| **Eligible studies synthesized narratively** | 2 |
| **Conference abstracts awaiting classification** | 1 |

The 405 forward-citation records reflect the corrected citation search restricted to eligible or included trial reports. The conference abstract by Waller (2025) remained awaiting classification because the available record did not report sufficient methods or results to determine eligibility.
