# Supplementary material for: Efficacy and safety of botulinum toxin type a for upper-limb essential tremor: a systematic review and meta-analysis of randomized placebo-controlled trials
Source: Front Neurol. 2026 Aug 28;17:1886329. doi: 10.3389/fneur.2026.1886329 (PMC13561811; doi:10.3389/fneur.2026.1886329)
Supplement: Supplementary file 2 [file Table_2.DOCX]

Supplementary Table S2. Reports excluded after detailed eligibility assessment and report awaiting classification

| Excluded report / identifier (n=5) | **Design or clinical topic** | **Primary reason for exclusion** | **PICOS domain** | **Clarification** |
| --- | --- | --- | --- | --- |
| **Pahwa et al. (1995), “Botulinum toxin treatment of essential head tremor”** | Randomized double-blind placebo-controlled crossover study of essential head tremor | Anatomical treatment target did not meet the final upper-limb criterion | Participants / anatomical target | Cervical-muscle injections for head tremor; no separately extractable upper-limb ET data. |
| **Marques et al. (2023), “Trial of Botulinum Toxin for Isolated or Essential Head Tremor”** | Multicentre randomized placebo-controlled trial of isolated or essential head tremor | Population and anatomical treatment target were ineligible | Participants / anatomical target | Enrolled isolated or essential head tremor and treated splenius capitis muscles; upper-limb ET was not evaluated. |
| **Asadi et al. (2023), ultrasound-guided BoNT-A versus oral propranolol** | Clinical trial in moderate-to-severe essential hand tremor | Ineligible active comparator | Comparator | No placebo or saline group; comparative-effectiveness question versus propranolol. |
| **Guglielmino et al. (2018), BoNT versus propranolol for essential/dystonic vocal tremor** | Study of vocal tremor with an active comparator | Ineligible comparator/intervention question | Comparator / participants | Compared BoNT with propranolol and addressed vocal rather than upper-limb tremor. |
| **NCT02909907 (FLEX-D ET), flexor-plus-extensor versus flexor-only BoNT injections** | Randomized comparison of two BoNT injection patterns | Ineligible comparator/intervention question | Comparator / intervention | No overall BoNT-A versus placebo comparison. |

Of 16 reports retrieved and assessed for eligibility, five were excluded and one remained awaiting classification. The remaining 10 reports were linked to six included studies (Supplementary Table S3). One primary exclusion reason was assigned to each excluded report for PRISMA accounting. BoNT-A, botulinum toxin type A; ET, essential tremor; PICOS, Participants, Intervention, Comparator, Outcomes and Study design.

Report awaiting classification (n=1)

| Report awaiting classification | **Available information** | **Status** | **Reason** |
| --- | --- | --- | --- |
| **Waller N. “Efficacy of Botulinum Toxin Type A for Refractory Upper Limb Essential Tremors” (conference abstract, 2025)** | Title and meeting record only; no study design, sample size, comparator, outcomes or results were available. | Awaiting classification | Insufficient information to determine whether the report describes an eligible randomized placebo-controlled trial. |
