# Supplementary material for: Efficacy and safety of botulinum toxin type a for upper-limb essential tremor: a systematic review and meta-analysis of randomized placebo-controlled trials
Source: Front Neurol. 2026 Aug 28;17:1886329. doi: 10.3389/fneur.2026.1886329 (PMC13561811; doi:10.3389/fneur.2026.1886329)
Supplement: Supplementary file 3 [file Table_3.DOCX]

Supplementary Table S3. Study-to-report linkage and source-level result extraction for included studies

**Purpose.** This table provides an auditable link from each included study to the numerical data and public sources used in the review. One principal eligibility report was counted per included study in Figure 1; linked registry, conference, and sponsor sources used only for verification or narrative supplementation were not counted as separate eligibility reports. Article locations refer to published page, table, or figure numbers; registry locations refer to the named endpoint or adverse-event section.

Part A. Linkage of included studies to principal reports and additional public data sources

| **Study ID** | **Report or public source** | **Source type and PRISMA role** | **Information verified and contribution to review** |
| --- | --- | --- | --- |
| Brin 2001 | Brin MF, Lyons KE, Doucette J, et al. Neurology. 2001;56:1523–1528. doi:10.1212/WNL.56.11.1523 | Peer-reviewed article; Principal included report counted in Figure 1. | Design, randomization, dose groups, Week-6 tremor/global-response data, grip strength, and adverse reactions. Quantitative synthesis; 50-U and 100-U arms combined once against the shared placebo group. |
| Jankovic 1996 | Jankovic J, Schwartz K, Clemence W, et al. Mov Disord. 1996;11:250–256. doi:10.1002/mds.870110306 | Peer-reviewed article; Principal included report counted in Figure 1. | Randomized Week-4 outcomes before rescue dosing, examiner/subject global response, functional score, responder data, and weakness. Global-response meta-analyses; responder result used only in an exploratory conversion; function and safety summarized separately. |
| Mittal 2018 | Mittal SO, Machado D, Richardson D, et al. Parkinsonism Relat Disord. 2018;56:65–69. doi:10.1016/j.parkreldis.2018.06.019 | Peer-reviewed article; Principal included report counted in Figure 1. | Crossover design, Week-4/Week-8 FTM and NIHCGC analyses, PGIC, weakness, withdrawal, follow-up, and carryover methods. Narrative synthesis only; no paired effect with variance, paired binary table, sufficient participant-level paired data, or separable first-period data. |
| Jog 2020 | Jog M, Lee J, Scheschonka A, et al. Toxins (Basel). 2020;12:807. doi:10.3390/toxins12120807 | Peer-reviewed article; Principal included report counted in Figure 1. | Randomization, kinematic targeting, FTM, physician/patient GICS, grip strength, device outcomes, treatment-related TEAEs, SAEs, weakness, and discontinuation through Week 24. Quantitative synthesis for eligible efficacy and safety outcomes; adjusted function/device results summarized separately. |
| Jog 2020 | ClinicalTrials.gov. NCT02207946. https://clinicaltrials.gov/study/NCT02207946 | Trial-registry record; Linked verification source; not counted as a separate report in Figure 1. | Prospective design and outcome-definition verification. Supporting report linked to Jog 2020; not counted as a separate study. |
| NCT04766723 | ClinicalTrials.gov. NCT04766723. https://clinicaltrials.gov/study/NCT04766723 | Trial-registry record; Linked verification source; not counted as a separate report in Figure 1. | Eligibility, intervention, treatment periods, prespecified outcomes, and study status. Linked to the official EudraCT results record; randomized unilateral Cycle 1 only was eligible for comparative synthesis. |
| NCT04766723 | EU Clinical Trials Register. EudraCT 2021-001988-24, results version 3 (published 27 May 2026). https://www.clinicaltrialsregister.eu/ctr-search/trial/2021-001988-24/results | Official results record; Principal included report counted in Figure 1. | Randomized/treated/evaluable denominators; Week-6 TETRAS and GICS results; Week-24 treatment-related TEAEs, muscular weakness, SAEs, and discontinuation. Quantitative synthesis for eligible unilateral Cycle-1 results. Exact endpoint titles and result sections are listed in Part B. |
| ELATE / NCT05216250 | ClinicalTrials.gov. NCT05216250. https://clinicaltrials.gov/study/NCT05216250 | Trial-registry record; Principal included report counted in Figure 1. | Phase 2 design, eligibility, planned/registered enrollment, treatment cycles, and prespecified outcomes. Narrative repeated-treatment evidence. |
| ELATE / NCT05216250 | Patterson K, Alibhai N, Khosla D, James L. Mov Disord. 2024;39(Suppl 1):Abstract 1551; Reference 19 in the main manuscript. | Conference design abstract; Linked conference source; not counted as a separate report in Figure 1. | Eligibility, treatment schedule, planned sample, and outcome framework. Narrative supporting source; no numerical result dataset. |
| ELATE / NCT05216250 | AbbVie. Positive topline results from the Phase 2 ELATE trial. News release, 6 October 2025; Reference 20 in the main manuscript. | Official sponsor results release; Linked sponsor source; not counted as a separate report in Figure 1. | TREDS-R group changes, P value, muscular-weakness percentages, repeated-treatment timing, and presentation context. Narrative emerging evidence only; denominators, variance estimates, and a complete peer-reviewed result report were unavailable. |

Part B1. Continuous efficacy and grip-strength inputs used in quantitative syntheses

| **Analysis / study / time point / measure** | **Exact source location** | **BoNT-A analysis data** | **Placebo analysis data** | **Data handling / derivation** | **Entered estimate / role** |
| --- | --- | --- | --- | --- | --- |
| P1 Clinician-rated tremor severity Brin 2001; Week 6; postural tremor rating (0–4) | Brin article, p.1525, Table 1, “Postural—6 wk” | 50 U: 1.7 (0.8; n=40) 100 U: 1.5 (0.8; n=42) Combined: 1.598 (0.801; n=82) | 2.1 (0.6; n=44) | Active-arm mean and SD combined using the Cochrane shared-control formula. Lower scores indicate less tremor. | Hedges g −0.68 (95% CI −1.05 to −0.30); primary P1. |
| P1 Clinician-rated tremor severity Jog 2020; Week 4; FTM tremor score for injected limb | Jog article, Section 2.2 and Figure 2A | Change −1.9 (1.9; n=18) | Change −0.5 (1.8; n=11) | Observed-case change scores; lower values indicate improvement. | Hedges g −0.73 (95% CI −1.51 to 0.05); primary P1. |
| P1 Clinician-rated tremor severity NCT04766723; Week 6; TETRAS Performance dominant upper-limb score | EudraCT v3, End Points: “Change From Baseline to Week 6 in TETRAS Performance Dominant UL Score” | Change −2.3 (2.60; n=50) | Change −0.9 (1.56; n=26) | Official Cycle-1 FAS-UP arithmetic mean change; lower values indicate improvement. | Hedges g −0.60 (95% CI −1.09 to −0.12); primary P1. |
| S2 Investigator-rated global response Brin 2001; Week 6; investigator subjective assessment (−4 to +4) | Brin article, p.1525, Table 1, “Subjective assessment by investigator—6 wk” | 50 U: 0.5 (0.7; n=40) 100 U: 0.5 (0.9; n=42) Combined input: 0.500 (0.81; n=82) | 0.0 (0.6; n=44) | Active arms combined. Scores were relative to the preceding visit, not baseline; positive values indicate improvement. | Hedges g 0.67 (95% CI 0.29 to 1.04); retained in primary S2; exclusion tested in sensitivity analysis. |
| S2 Investigator-rated global response Jankovic 1996; Week 4; examiner treatment-response rating | Jankovic article, p.253, Table 2, “Treatment response rating (examiner)” | 2.00 (0.95; n=12) | 0.55 (1.04; n=11) | Clean randomized comparison before rescue dosing; positive values indicate improvement. | Hedges g 1.41 (95% CI 0.47 to 2.34); primary S2. |
| S2 Investigator-rated global response Jog 2020; Week 4; physician-rated GICS | Jog article, Section 2.3 and Supplementary Figure S1 | 0.8 (0.7; n=18) | 0.1 (0.7; n=11) | Observed cases; higher values indicate improvement. | Hedges g 0.97 (95% CI 0.18 to 1.77); primary S2. |
| S2 Investigator-rated global response NCT04766723; Week 6; investigator GICS | EudraCT v3, End Points: “Investigator’s GICS Score of Motor-dominant UL at Week 6” | 1.2 (1.36; n=50) | 0.8 (1.03; n=26) | Official Cycle-1 FAS-UP arithmetic mean; higher values indicate improvement. | Hedges g 0.31 (95% CI −0.16 to 0.79); primary S2. |
| S3 Patient-rated global response Brin 2001; Week 6; subject subjective assessment (−4 to +4) | Brin article, p.1525, Table 1, “Subjective assessment by subject—6 wk” | 50 U: 0.4 (0.9; n=40) 100 U: 0.5 (1.0; n=42) Combined: 0.451 (0.948; n=82) | 0.0 (0.8; n=44) | Active arms combined. Scores were relative to the preceding visit; positive values indicate improvement. | Hedges g 0.50 (95% CI 0.13 to 0.87); retained in primary S3; exclusion tested in sensitivity analysis. |
| S3 Patient-rated global response Jankovic 1996; Week 4; subject treatment-response rating | Jankovic article, p.253, Table 2, “Treatment response rating (subject)” | 1.83 (1.27; n=12) | 0.73 (1.27; n=11) | Clean randomized comparison before rescue dosing; positive values indicate improvement. | Hedges g 0.83 (95% CI −0.03 to 1.69); primary S3. |
| S3 Patient-rated global response Jog 2020; Week 4; patient-rated GICS | Jog article, Section 2.3 and Supplementary Table S1 | 0.6 (0.6; n=18) | 0.3 (0.6; n=11) | Observed cases; higher values indicate improvement. | Hedges g 0.49 (95% CI −0.28 to 1.25); primary S3. |
| S3 Patient-rated global response NCT04766723; Week 6; subject GICS | EudraCT v3, End Points: “Subject’s Global Impression of Change Scale Score of Motor-dominant UL at Week 6” | 0.9 (1.27; n=50) | 0.8 (1.14; n=26) | Official Cycle-1 FAS-UP arithmetic mean; higher values indicate improvement. | Hedges g 0.08 (95% CI −0.39 to 0.55); primary S3. |
| S4 Maximum grip strength Brin 2001; Week 6; first dynamometer measurement | Brin article, p.1526, Table 2, “Grip strength (first measurement)—6 wk” | 50 U: 27.3 (10.9; n=40) 100 U: 21.2 (10.7; n=42) Combined: 24.176 (11.161; n=82) | 32.2 (14.5; n=44) | Active-arm endpoint means and SDs combined; kilograms. | MD −8.02 kg (95% CI −12.94 to −3.11); primary S4. |
| S4 Maximum grip strength Jog 2020; Week 4; maximum grip strength | Jog article, Section 2.5 and Figure 4 | 23.32 (12.04; n=19) kg | 27.64 (11.58; n=11) kg | Week-4 endpoint means selected; no change-score substitution. | MD −4.32 kg (95% CI −13.05 to 4.41); primary S4. |

Part B2. Binary safety inputs used in quantitative syntheses

| **Analysis / study / time point / measure** | **Exact source location** | **BoNT-A analysis data** | **Placebo analysis data** | **Data handling / derivation** | **Entered estimate / role** |
| --- | --- | --- | --- | --- | --- |
| P2 Muscular weakness Jog 2020; through Week 24; localized muscular weakness | Jog article, Section 2.5 and Supplementary Table S2 | 2/19 | 0/11 | Participants with at least one event; safety evaluation set. | RD 0.11 (95% CI −0.08 to 0.29); primary P2. |
| P2 Muscular weakness NCT04766723; through Week 24; muscular weakness | EudraCT v3, Adverse Events: “Muscular weakness” (unilateral period) | 12/50 | 0/27 | Subjects affected/exposed in unilateral Cycle 1; treated safety population. | RD 0.24 (95% CI 0.11 to 0.37); primary P2. |
| S5 Treatment-related TEAEs Jog 2020; through Week 24; ≥1 treatment-related TEAE | Jog article, Section 2.5 and Supplementary Table S2 | 3/19 | 1/11 | Participants with at least one treatment-related event. | RD 0.07 (95% CI −0.17 to 0.30); S5. |
| S5 Treatment-related TEAEs NCT04766723; through Week 24; ≥1 treatment-related TEAE | EudraCT v3, End Points: “Percentage of Subjects With At-least One Treatment Related TEAE” | 15/50 (30.0%) | 1/27 (3.7%) | Counts derived exactly from reported percentages and denominators (30.0% of 50; 3.7% of 27). | RD 0.26 (95% CI 0.12 to 0.41); S5. |
| S6 Serious adverse events Jog 2020; through Week 24; SAE | Jog article, Section 2.5 | 0/19 | 2/11 | Participants with ≥1 SAE; both placebo SAEs reported as unrelated to study medication. | RD −0.18 (95% CI −0.42 to 0.06); S6. |
| S6 Serious adverse events NCT04766723; through Week 24; SAE | EudraCT v3, Adverse Events: “Total subjects affected by serious adverse events” (unilateral period) | 3/50 | 2/27 | Subjects affected/exposed in unilateral Cycle 1. | RD −0.01 (95% CI −0.13 to 0.10); S6. |
| S7 Discontinuation due to adverse events Jog 2020; through Week 24 | Jog article, Figure 1 and Section 2.5 | 0/19 | 1/11 | Placebo participant discontinued after chest discomfort, considered unrelated to study medication. | RD −0.09 (95% CI −0.29 to 0.11); S7. |
| S7 Discontinuation due to adverse events NCT04766723; through Week 24 | EudraCT v3, Subject Disposition, unilateral period: “Adverse event, non-fatal” | 1/51 | 0/27 | Randomized denominator used for withdrawal outcome; one active-group non-fatal AE discontinuation. | RD 0.02 (95% CI −0.05 to 0.09); S7. |

Part C. Sensitivity analyses and outcomes summarized separately or narratively

| **Outcome / study / time point** | **Exact source location** | **BoNT-A result** | **Placebo result** | **Derivation / between-group result** | **Reason for separate or narrative treatment** |
| --- | --- | --- | --- | --- | --- |
| P1 sensitivity: alternative tremor measure Brin 2001; Week 6; kinetic tremor | Brin article, p.1525, Table 1, “Kinetic—6 wk” | 50 U: 1.6 (0.7; n=40) 100 U: 1.7 (0.8; n=42) Combined: 1.651 (0.750; n=82) | 2.2 (0.7; n=44) | Hedges g −0.74 (95% CI −1.12 to −0.37); substituted for Brin postural result. | Alternative eligible Brin measurement; pooled sensitivity estimate −0.70 (95% CI −0.97 to −0.42). |
| P1 exploratory responder conversion Jankovic 1996; Week 4; ≥1-grade postural-tremor improvement | Jankovic article, p.253, Table 2 | 11/12 responders | 4/11 responders | OR=(11×7)/(1×4)=19.25; approximate SMD=ln(OR)×√3/π=1.63; sign reversed so negative favours BoNT-A; SE=0.672. | Binary responder outcome transformed to continuous scale; exploratory only. |
| S4 sensitivity: alternative grip measurement Brin 2001; Week 6; second dynamometer measurement | Brin article, p.1526, Table 2, “Grip strength (second measurement)—6 wk” | 50 U: 27.2 (11.0; n=40) 100 U: 21.4 (10.4; n=42) Combined: 24.229 (11.023; n=82) | 33.0 (14.8; n=44) | MD −8.77 kg (95% CI −13.75 to −3.79); substituted for first measurement. | Alternative eligible Brin grip measurement; pooled sensitivity estimate −7.68 kg (95% CI −12.00 to −3.35). |
| Function Jankovic 1996; Week 4; UTRA tremor functional score | Jankovic article, p.253, Table 2 | Change −2.75 (4.47; n=12) | Change −3.18 (4.02; n=11) | Unadjusted MD 0.43 (95% CI −3.04 to 3.90). | Different functional construct; no clear between-group difference. |
| Function Jog 2020; Week 4; FTM Part B motor performance | Jog article, Section 2.2 and Figure 2B | Simple group mean not reported for selected adjusted analysis | Simple group mean not reported for selected adjusted analysis | Adjusted LS MD −2.30 (95% CI −3.80 to −0.90). | Adjusted ANCOVA estimand differed from other function/ADL outcomes. |
| ADL NCT04766723; Week 6; TETRAS ADL upper-limb score | EudraCT v3, End Points: “Change From Baseline to Week 6 in TETRAS Performance ADL UL Score” | Change −3.70 (5.00; n=50) | Change −3.80 (4.46; n=26) | Unadjusted MD 0.10 (95% CI −2.10 to 2.30). | Patient-reported ADL construct differed from the other functional estimands. |
| Functional impact NCT04766723; Week 6; TETRAS ADL Functional Impact score | EudraCT v3, End Points: “Change From Baseline to Week 6 in TETRAS ADL Functional Impact Score” | Change −1.20 (1.87; n=50) | Change −1.30 (1.83; n=26) | Unadjusted MD 0.10 (approximate 95% CI −0.77 to 0.97). | Trial-specific functional-impact construct; reported separately. |
| Device-measured tremor Jog 2020; Week 4; log-transformed accelerometric hand-tremor amplitude | Jog article, Section 2.4 and Figure 3B | Adjusted change incorporated in model | Adjusted change incorporated in model | LS MD −0.66 (95% CI −1.09 to −0.23). | Device, unit, and adjusted model differed from clinician-rated tremor outcomes. |
| Supportive safety: weakness Jankovic 1996; Week 4; mild/moderate finger weakness | Jankovic article, p.253, safety-results text | 11/12 | 0/11 | No pooled effect in the 24-week primary safety analysis. | Earlier follow-up and unusually high weakness frequency; summarized separately. |
| Supportive safety: weakness Brin 2001; through Week 16; hand weakness | Brin article, p.1526, adverse-reactions text | 44/88 (13/43 at 50 U; 31/45 at 100 U) | NR/45 | No comparative effect calculated; placebo event count was not reported and was not imputed as zero. | Could not enter comparative meta-analysis. |
| Crossover efficacy Mittal 2018; Week 4; paired FTM change | Mittal article, Results and Table 2 | Paired period comparison favoured incobotulinumtoxinA | Placebo period served as within-participant control | Wilcoxon signed-rank P<0.003. | No paired effect estimate with variance, participant-level paired data, or separable first-period data. |
| Crossover patient response Mittal 2018; PGIC across periods | Mittal article, Results | “Much improved”: 10/19 periods | “Much improved”: 3/19 periods | P=0.031; paired discordant cells not reported. | No valid paired binary effect calculable; narrative synthesis only. |
| Crossover weakness Mittal 2018; weakness across periods | Mittal article, Results | Mild weakness: 6 periods | Mild weakness: 4 periods | P=0.728; one participant withdrew because of unacceptable weakness. | Paired discordant cells not reported; narrative synthesis only. |
| Repeated-treatment disability ELATE/NCT05216250; primary assessment across Weeks 15, 18 and 21 after two blinded cycles; TREDS-R | AbbVie sponsor release, 6 Oct 2025, primary-endpoint results paragraph and “About the ELATE Study” | LS mean change −2.61 | LS mean change −1.61 | P=0.029; variance and analysis denominators not publicly reported. | Narrative emerging evidence only; insufficient traceable data for meta-analysis. |
| Repeated-treatment safety ELATE/NCT05216250; muscular weakness | AbbVie sponsor release, 6 Oct 2025, safety-results paragraph | 24.5% | 2.3% | Event counts and analysis denominators not reported; no count inferred. | Narrative emerging evidence only; complete peer-reviewed safety dataset unavailable. |

**Verification and derivation notes.** Randomized group sizes and outcome-specific analysis denominators are distinguished. Shared placebo groups were entered once. Brin active arms were combined using standard Cochrane formulas. Brin placebo weakness was not reported and was not coded as zero. Mittal 2018 was retained in the review but excluded from all meta-analyses because paired effects, paired binary tables, and separable first-period data were unavailable. The Jankovic responder conversion was exploratory and used SMD = ln(OR) × √3/π, with direction harmonized so negative values favoured BoNT-A. ELATE was summarized narratively and did not contribute to pooled estimates or GRADE participant counts.
