# Supplementary material for: Efficacy and safety of botulinum toxin type a for upper-limb essential tremor: a systematic review and meta-analysis of randomized placebo-controlled trials
Source: Front Neurol. 2026 Aug 28;17:1886329. doi: 10.3389/fneur.2026.1886329 (PMC13561811; doi:10.3389/fneur.2026.1886329)
Supplement: Supplementary file 4 [file Table_4.DOCX]

**Supplementary Table S4. Sensitivity analyses and outcomes not included in pooled estimates**

**Part A. Sensitivity analyses**

| **Outcome** | **Sensitivity scenario** | **Studies included** | **Effect measure and model** | **Effect estimate (95% CI)** | **I²** | **Interpretation** |
| --- | --- | --- | --- | --- | --- | --- |
| **Clinician-rated upper-limb tremor severity** | Alternative Brin kinetic-tremor measure substituted for postural tremor | Brin 2001; Jog 2020; NCT04766723 | Hedges’ g; random-effects, Wald CI | −0.70 (−0.97 to −0.42) | 0% | Direction and magnitude were consistent with the primary analysis. |
| **Clinician-rated upper-limb tremor severity** | Brin 2001 excluded | Jog 2020; NCT04766723 | Hedges’ g; random-effects, Wald CI | −0.64 (−1.05 to −0.23) | 0% | The result remained favourable despite fewer studies. |
| **Clinician-rated upper-limb tremor severity** | Exploratory addition of converted Jankovic responder result | Brin 2001; Jankovic 1996; Jog 2020; NCT04766723 | Generic inverse-variance SMD; random-effects, Wald CI | −0.70 (−0.97 to −0.43) | 0% | Direction was unchanged; exploratory because one binary result was converted to SMD. |
| **Investigator-rated global response** | Brin 2001 excluded | Jankovic 1996; Jog 2020; NCT04766723 | Hedges’ g; REML with modified Hartung–Knapp CI | 0.80 (−0.63 to 2.23) | 60.4% | Point estimate remained favourable, but precision decreased and the CI crossed the null. |
| **Patient-rated global response** | Brin 2001 excluded | Jankovic 1996; Jog 2020; NCT04766723 | Hedges’ g; REML with modified Hartung–Knapp CI | 0.36 (−0.63 to 1.34) | 21.1% | Point estimate remained favourable, but the CI crossed the null. |
| **Maximum grip strength** | Alternative Brin Week-6 dynamometer measurement | Brin 2001; Jog 2020 | Mean difference, kg; random-effects, Wald CI | −7.68 (−12.00 to −3.35) | 0% | Direction and magnitude were consistent with the primary analysis. |

**Part B. Functional, device-measured and narrative-only outcomes not pooled**

| **Study / outcome** | **Time point** | **BoNT-A result** | **Placebo result** | **Between-group result** | **Reason not pooled / interpretation** |
| --- | --- | --- | --- | --- | --- |
| **Jankovic 1996: UTRA functional score** | Week 4 | Change −2.75 (SD 4.47; n=12) | Change −3.18 (SD 4.02; n=11) | Unadjusted MD 0.43 (95% CI −3.04 to 3.90) | Different functional construct; no clear between-group difference. |
| **Jog 2020: FTM Part B motor performance** | Week 4 | Not reported as a simple group mean | Not reported as a simple group mean | Adjusted MD −2.30 (95% CI −3.80 to −0.90) | Adjusted ANCOVA estimand differed from other functional outcomes; favoured BoNT-A. |
| **NCT04766723: TETRAS ADL upper-limb score** | Week 6 | Change −3.70 (SD 5.00; n=50) | Change −3.80 (SD 4.46; n=26) | Unadjusted MD 0.10 (95% CI −2.10 to 2.30) | Different patient-reported construct; no clear between-group difference. |
| **NCT04766723: trial-specific functional-impact measure** | Week 6 | Change −1.20 (SD 1.87; n=50) | Change −1.30 (SD 1.83; n=26) | Unadjusted MD 0.10 (approx. 95% CI −0.77 to 0.97) | Trial-specific measure; no clear between-group difference. |
| **Jog 2020: log-transformed accelerometric tremor amplitude** | Week 4 | Adjusted change incorporated in model | Adjusted change incorporated in model | Least-squares MD −0.66 (95% CI −1.09 to −0.23) | Device, unit and model differed; favoured BoNT-A. |
| **Mittal 2018: FTM tremor score** | Week 4 of each crossover period | Paired period comparison favoured incobotulinumtoxinA | Placebo period served as the paired control | Wilcoxon signed-rank P<0.003 | No paired effect with variance or separable first-period data; narrative synthesis only. |
| **Mittal 2018: PGIC and weakness** | Across crossover periods | PGIC “much improved”: 10 periods; mild weakness: 6 periods | PGIC “much improved”: 3 periods; mild weakness: 4 periods | PGIC P=0.031; no paired binary effect calculable | Discordant paired cells were not reported; one withdrawal due to unacceptable weakness. |
| **ELATE / NCT05216250: TREDS-R after repeated treatment** | Week 18 | Sponsor-reported LS mean change −2.61 | Sponsor-reported LS mean change −1.61 | P=0.029; muscular weakness 24.5% vs 2.3% | Registry/sponsor/conference-level evidence; unresolved enrollment discrepancy and no complete peer-reviewed dataset. |

Negative standardized effects favour BoNT-A for clinician-rated tremor severity; positive standardized effects favour BoNT-A for global response; negative grip-strength differences indicate lower strength with BoNT-A. The Jankovic responder result was converted using SMD = ln(OR) × √3/π and used only in an exploratory sensitivity analysis. ADL, activities of daily living; ANCOVA, analysis of covariance; CI, confidence interval; FTM, Fahn–Tolosa–Marín; LS, least squares; MD, mean difference; PGIC, Patient Global Impression of Change; REML, restricted maximum likelihood; SD, standard deviation; SMD, standardized mean difference; TETRAS, The Essential Tremor Rating Assessment Scale; TREDS-R, Tremor Disability Scale-Revised; UTRA, Unified Tremor Rating Assessment.
