# Supplementary material for: Efficacy and safety of botulinum toxin type a for upper-limb essential tremor: a systematic review and meta-analysis of randomized placebo-controlled trials
Source: Front Neurol. 2026 Aug 28;17:1886329. doi: 10.3389/fneur.2026.1886329 (PMC13561811; doi:10.3389/fneur.2026.1886329)
Supplement: Supplementary file 5 [file Table_5.DOCX]

**Supplementary Table S5. Complete GRADE evidence profile**

*Population: adults with upper-limb essential tremor | Intervention: botulinum toxin type A injection | Comparator: placebo or saline*

| **Outcome / follow-up** | **Studies** | **Risk of bias** | **Inconsistency** | **Indirectness** | **Imprecision** | **Publication bias / other** | **BoNT-A** | **Placebo** | **Effect estimate (95% CI)** | **Certainty** | **Importance** | **Footnote** |
| --- | --- | --- | --- | --- | --- | --- | --- | --- | --- | --- | --- | --- |
| **Clinician-rated upper-limb tremor severity; 4–6 weeks** | 3 | Serious | Not serious | Not serious | Not serious | Not downgraded; bias could not be ruled out | 150 participants | 81 participants | SMD 0.66 SD lower (0.94 lower to 0.38 lower) | Moderate ⊕⊕⊕◯ | Critical | a |
| **Muscular weakness; through Week 24** | 2 | Serious | Not serious | Not serious | Serious | Not downgraded; sparse evidence | 14/69 | 0/38 | RD 0.19 (0.06 to 0.32); 190 more per 1,000 | Low ⊕⊕◯◯ | Critical | b |
| **Investigator-rated global response; 4–6 weeks** | 4 | Serious | Not serious | Not serious | Serious | Not downgraded; few studies | 162 participants | 92 participants | SMD 0.70 SD higher (0.06 to 1.34 higher) | Low ⊕⊕◯◯ | Important | c |
| **Patient-rated global response; 4–6 weeks** | 4 | Serious | Not serious | Not serious | Serious | Not downgraded; few studies | 162 participants | 92 participants | SMD 0.40 SD higher (0.14 to 0.67 higher) | Low ⊕⊕◯◯ | Critical | d |
| **Maximum grip strength; 4–6 weeks** | 2 | Serious | Not serious | Not serious | Serious | Not downgraded; few studies | 101 participants | 55 participants | MD 7.13 kg lower (11.42 lower to 2.85 lower) | Low ⊕⊕◯◯ | Important | e |
| **Treatment-related TEAEs; through Week 24** | 2 | Serious | Not serious | Not serious | Very serious | Not downgraded; sparse events | 18/69 | 2/38 | RD 0.19 (0.00 to 0.37); 190 more per 1,000 | Very low ⊕◯◯◯ | Critical | f |
| **Serious adverse events; through Week 24** | 2 | Not serious | Not serious | Not serious | Very serious | Not downgraded; very sparse events | 3/69 | 4/38 | RD −0.07 (−0.22 to 0.09); 70 fewer per 1,000 | Low ⊕⊕◯◯ | Critical | g |
| **Discontinuation due to adverse events; through Week 24** | 2 | Not serious | Not serious | Not serious | Very serious | Not downgraded; very sparse events | 1/70 | 1/38 | RD −0.01 (−0.11 to 0.10); 10 fewer per 1,000 | Low ⊕⊕◯◯ | Important | h |

GRADE certainty: High ⊕⊕⊕⊕; Moderate ⊕⊕⊕◯; Low ⊕⊕◯◯; Very low ⊕◯◯◯. Publication bias was not formally assessed because each synthesis contained too few studies; the domain was not downgraded, but publication bias could not be excluded. RD, risk difference; SMD, standardized mean difference; MD, mean difference; TEAE, treatment-emergent adverse event.

**Downgrading explanations**

| **Footnote** | **Explanation** |
| --- | --- |
| **a** | Downgraded one level for risk of bias because all pooled clinician-rated results had some concerns, particularly possible functional unblinding and selective result choice. Mittal 2018 was narrative only and did not contribute to the pooled effect. |
| **b** | Downgraded one level for risk of bias and one level for imprecision because only 14 weakness events occurred and the optimal information size was not met. Mittal 2018 lacked paired binary information and was narrative only. |
| **c** | Downgraded one level for risk of bias because two subjective investigator-rated results were at high risk, and one level for imprecision because the main and sensitivity intervals crossed review-specific decision thresholds. |
| **d** | Downgraded one level for risk of bias because two subjective patient-rated results were at high risk, and one level for imprecision because confidence intervals crossed the review-specific important-effect threshold. Mittal PGIC data were narrative only. |
| **e** | Downgraded one level for risk of bias because grip strength was effort-dependent and multiple measurements or analyses were available, and one level for imprecision because the interval included both an important and a smaller reduction. |
| **f** | Downgraded one level for risk of bias and two levels for very serious imprecision because events were sparse and the interval included both no effect and important harm. |
| **g** | Downgraded two levels for very serious imprecision because events were sparse and the interval included important benefit, no effect and important harm. |
| **h** | Downgraded two levels for very serious imprecision because only two withdrawals occurred and the interval included important benefit, no effect and important harm. |

Narrative evidence: Mittal 2018 was an eligible randomized crossover trial but did not contribute to pooled effects because paired effect estimates with appropriate variance, paired binary tables and separable first-period data were unavailable. ELATE/NCT05216250 was treated as emerging repeated-treatment evidence and was not included in pooled GRADE rows.
